# Supplementary material for: The SNP-Based Profiling of Montecristo Feral Goat Populations Reveals a History of Isolation, Bottlenecks, and the Effects of Management
Source: Genes (Basel). 2022 Jan 24;13(2):213. doi: 10.3390/genes13020213 (PMC8872249; doi:10.3390/genes13020213)
Supplement: Supplementary file 1 [file genes-13-00213-s001.zip › STable1.pdf]

Breed acronym	Breed name	Country of Origin	Source Dataset	N of animals Raw dataset	N of animals Working Dataset	H_O	H_E	F_{IS}
ALP_IT	Alpine	Italy	AdaptMap	160	30	0.399	0.405	0.025
ANK	Ankara	Turkey	AdaptMap	20	20	0.398	0.397	0.006
ARG	Argentata dell'Etna	Italy	IGC2	48	29	0.416	0.413	0.008
ASP	Capra dell'Aspromonte	Italy	IGC2	24	24	0.402	0.406	0.026
BAR	Barcha	Morocco	AdapMap	4	4	0.391	0.395	0.038
BEY	Bermeya	Spain	AdaptMap	24	24	0.404	0.397	-0.006
BEZ	Bezoar	Iran	AdaptMap	7	7	0.281	0.376	0.233***
BIA	Bianca Monticellana	Italy	IGC2	24	24	0.393	0.403	0.041
BIO	Bionda dell'Adamello	Italy	IGC2	24	24	0.404	0.405	0.017
BRK	Barki	Egypt	AdaptMap	153	28	0.397	0.403	0.020
CAM	Camosciata	Italy	IGC2	143	29	0.406	0.404	0.005
CAP	Capestrina	Italy	IGC2	24	22	0.404	0.403	0.016
CRS	Corsa	France	AdaptMap	30	29	0.401	0.404	0.020
DDS	Derivata di Siria	Italy	IGGC2	32	30	0.382	0.402	0.060
DRA	Draa	Morocco	AdaptMap	4	4	0.387	0.394	0.035
FAC	Facciuta della Valnerina	Italy	IGC2	24	23	0.395	0.410	0.055
FSS	Fosses	France	AdaptMap	26	26	0.394	0.394	0.013
FUL	Fulva del Lazio	Italy	IGC2	22	22	0.415	0.411	0.009
GAR	Garganica	Italy	IGC2	40	27	0.403	0.403	0.011
GCI	Grigia Ciociara	Italy	IGC2	43	30	0.405	0.409	0.023
GHA	Ghazalia	Morocco	AdaptMap	4	4	0.386	0.395	0.044
GIR	Girgentana	Italy	IGC2	59	30	0.357	0.371	0.025
GRF	Garfagnana	Italy	IGC2	28	27	0.399	0.405	0.028
IRA	Iranian goat	Iran	AdaptMap	9	9	0.381	0.399	0.075
JON	Jonica	Italy	IGC2	16	11	0.417	0.388	-0.083
KIL	KIl	Turkey	AdaptMap	25	25	0.404	0.401	0.003
KLS	Kilis	Turkey	AdaptMap	40	30	0.401	0.400	0.006
LIV	Capra di Livo	Italy	IGC2	24	22	0.407	0.402	0.003
MAL	Maltese	Italy	AdaptMap	16	16	0.365	0.379	0.047
MES	Messinese	Italy	IGC2	24	22	0.413	0.409	0.008
MLG	Malaguena	Spain	AdaptMap	42	30	0.417	0.412	0.000
MNT_M	Montecristo	Italy	IGC2	18	17	0.325	0.377	-0.086
MNT_I	Montecristo	Italy	IGC2	32	30	0.272	0.347	-0.007
MON	Capra di Montefalcone	Italy	IGC2	24	23	0.403	0.408	0.029
MOR	Moroccan goat	Morocco	AdaptMap	10	10	0.380	0.396	0.068
MUG	Murciano-granadina	Spain	AdaptMap	20	20	0.402	0.397	0.001
NDA	Noire de l'Atlas	Morocco	AdaptMap	4	4	0.384	0.396	0.055
NIC	Nicastrse	Italy	IGC2	24	24	0.396	0.408	0.045
NOR	Nord	Morocco	AdaptMap	4	4	0.394	0.398	0.059
NVE	Nera di Verzasca	Italy	IGC2	19	18	0.395	0.394	0.003

ORO	Orobica	Italy	IGC2	23	23	0.360	0.364	0.004
OSS	Oasis	Egypt	AdaptMap	72	30	0.373	0.395	0.056
PTV	Poitevine	France	AdaptMap	29	27	0.375	0.381	0.014
PVC	Provencale	France	AdaptMap	18	18	0.409	0.397	-0.015
PYR	Pyrenean	France	AdaptMap	27	27	0.374	0.388	0.041
RAS	Blanca de Rasquera	Spain	AdaptMap	20	20	0.378	0.388	0.036
RCC	Roccaverano	Italy	IGC2	28	28	0.401	0.416	0.052
RME	Rossa Mediterranea	Italy	IGC2	46	21	0.410	0.396	-0.038
SAA	Saanen	Italy	IGC2	44	30	0.416	0.410	-0.001
SAM	Maltese Sampled in Sardina	Italy	IGC2	15	14	0.375	0.371	-0.010
SAR	Sarda	Italy	IGC2	33	27	0.403	0.409	0.0261
SID	Saidi	Egypt	AdaptMap	60	30	0.384	0.400	0.044
TER	Capra di Teramo	Italy	IGC2	43	30	0.385	0.377	-0.016
TUN	Tunisian	Tunisia	AdapMap	23	23	0.401	0.401	0.015
VAL	Valdostana	Italy	IGC2	24	24	0.371	0.384	0.039
VLS	Vallesana	Italy	IGC2	24	23	0.348	0.370	0.051
VPS	Capra della Valpassiria	Italy	IGC2	24	24	0.402	0.407	0.030
TOT.				1871	1251	0.399	0.396	0.025
