# Supplementary material for: The SNP-Based Profiling of Montecristo Feral Goat Populations Reveals a History of Isolation, Bottlenecks, and the Effects of Management
Source: Genes (Basel). 2022 Jan 24;13(2):213. doi: 10.3390/genes13020213 (PMC8872249; doi:10.3390/genes13020213)
Supplement: Supplementary file 1 [file genes-13-00213-s001.zip › STable2.pdf]

Length category (Mb)	MNT_M	MNT_I
0-2	188	3209
2-4	252	2408
4-8	258	1155
8-16	217	348
>16	213	71
Tot.	1128	7191
