# Supplementary material for: The SNP-Based Profiling of Montecristo Feral Goat Populations Reveals a History of Isolation, Bottlenecks, and the Effects of Management
Source: Genes (Basel). 2022 Jan 24;13(2):213. doi: 10.3390/genes13020213 (PMC8872249; doi:10.3390/genes13020213)
Supplement: Supplementary file 1 [file genes-13-00213-s001.zip › STable3.pdf]

Run	Population	Chr	Start (bp)	End (bp)	genes
ROH	MNT_I	1	38552149	39474884	-
ROH	MNT_I	2	30528787	31463338	ENSCHIG00000006284, ENSCHIG00000006293, ENSCHIG00000006309, TNP1, SMARCAL1, MARCHF4
ROH	MNT_I	3	38397951	38432723	-
ROH	MNT_I	3	52713763	55922240	ST6GALNAC5
ROH	MNT_I	11	96495649	96534269	ENSCHIG00000004988
ROH	MNT_M	16	13996242	20982479	ENSCHIG00000000862, ENSCHIG00000000869, ENSCHIG00000000876, ENSCHIG00000000880, ENSCHIG00000000882, 5S_rRNA, RRP15, KCTD3, BRINP3, GPATCH2, ESRRG, ENSCHIG00000021102, TGFB2, SPATA17
HRR	MNT_I	6	23704450	24651938	PPP3CA, EMCN
HRR	MNT_I	6	92891918	93001162	CNOT6L
HRR	MNT_I	13	47213471	47526560	SHLD1, GPCPD1
HRR	MNT_M	1	105425339	105469270	OTOL1
HRR	MNT_M	2	89186937	89310361	LYPD6B
HRR	MNT_M	2	89822439	89891526	ENSCHIG00000000666
HRR	MNT_M	8	29737003	29990995	NFIB
HRR	MNT_M	8	69147795	69192956	BMP1
HRR	MNT_M	13	27167011	27654015	ENSCHIG00000000499, OPTN, BEND7, MCM10, PHYH
HRR	MNT_M	14	71387238	72223053	ASAP1, ADCY8
HRR	MNT_M	16	69226610	69455422	TATDN3, RPS6KC1, ANGEL2, NSL1
HRR	MNT_M	18	43777759	43932769	ENSCHIG00000004436, ENSCHIG00000004439
HRR	MNT_M	22	44176757	44243832	ENSCHIG00000015055
HRR	MNT_M	24	46431005	46854646	LOXHD1, KATNAL2, ST8SIA5, PIAS2
HRR	MNT_M	24	47100548	47587014	ENSCHIG00000002761, ENSCHIG00000002755, SKOR2
HRR	MNT_M	24	50751818	50783212	SMAD4
