# Supplementary material for: The SNP-Based Profiling of Montecristo Feral Goat Populations Reveals a History of Isolation, Bottlenecks, and the Effects of Management
Source: Genes (Basel). 2022 Jan 24;13(2):213. doi: 10.3390/genes13020213 (PMC8872249; doi:10.3390/genes13020213)
Supplement: Supplementary file 1 [file genes-13-00213-s001.zip › Supplementary_Captions.pdf]

***Supplementary Table 1**: Breed code, breed name, country of origin, source dataset (AdaptMap* [18]*; IGC2* [16]*), number of individuals pre-QC (Raw dataset)), number of individuals in the working dataset (WD), observed heterozygosity (H_O), expected heterozygosity (H_E) values corrected over the number of usable loci, inbreeding coefficient (F_{IS}). Statistical significance as follows: ***= P< 0.005.*

***Supplementary Figure 1:** Schematic representation of: (a) Scenario 1; (b) Scenario 2; (c) Scenario 3; and (d) Scenario 4 of ABC-RF anaysis. Populations are coded as follow: pop 1: CRS; pop 2: GRF; pop 3: MNT_M; pop 4: MNT_I; pop 5: SAR.*

***Supplementary Figure 2:** Supervised Principal Component Analysis (SPC1 vs. SPC2). The percentages of variance explained by each component are given into brackets.*

***Supplementary Figure 3:** Cross-Validation CV error values calculated for Admixture K values 2-45.*

***Supplementary Figure 4**: (a) The mean and standard deviation (SD) calculated across 5 iterations for the composite likelihood L(m) (left axis, black circles) and proportion of variance explained (right axis, red circles). (b) The second-order rate of change (Dm) across values of m.*

***Supplementary Figure 5**: Changes in estimated effective population size (Ne) along the last 1,000 generations.*

***Supplementary Figure 6**: Projection of the datasets from the training set on the first two linear discriminant analysis (LDA) axes. The location of the observed dataset in the LDA projection is indicated by the red dot.*

***Supplementary Table 2**: Summary of the number of ROH segments for different length categories scored in the two Montecristo goat nuclei.*

***Supplementary table 3:** Table summarising the 0.1% most shared regions of Runs of Homozygosity (ROH) and Heterozygosity Rich Regions (HRR) identified in the two Montecristo populations with the genes included.*

***Supplementary File 1**: ROHs of the two Montecristo populations plotted chromosome-wise.*
