# Supplementary material for: The SNP-Based Profiling of Montecristo Feral Goat Populations Reveals a History of Isolation, Bottlenecks, and the Effects of Management
Source: Genes (Basel). 2022 Jan 24;13(2):213. doi: 10.3390/genes13020213 (PMC8872249; doi:10.3390/genes13020213)
Supplement: Supplementary file 1 [file genes-13-00213-s001.zip › Supplementary_File1.pdf]

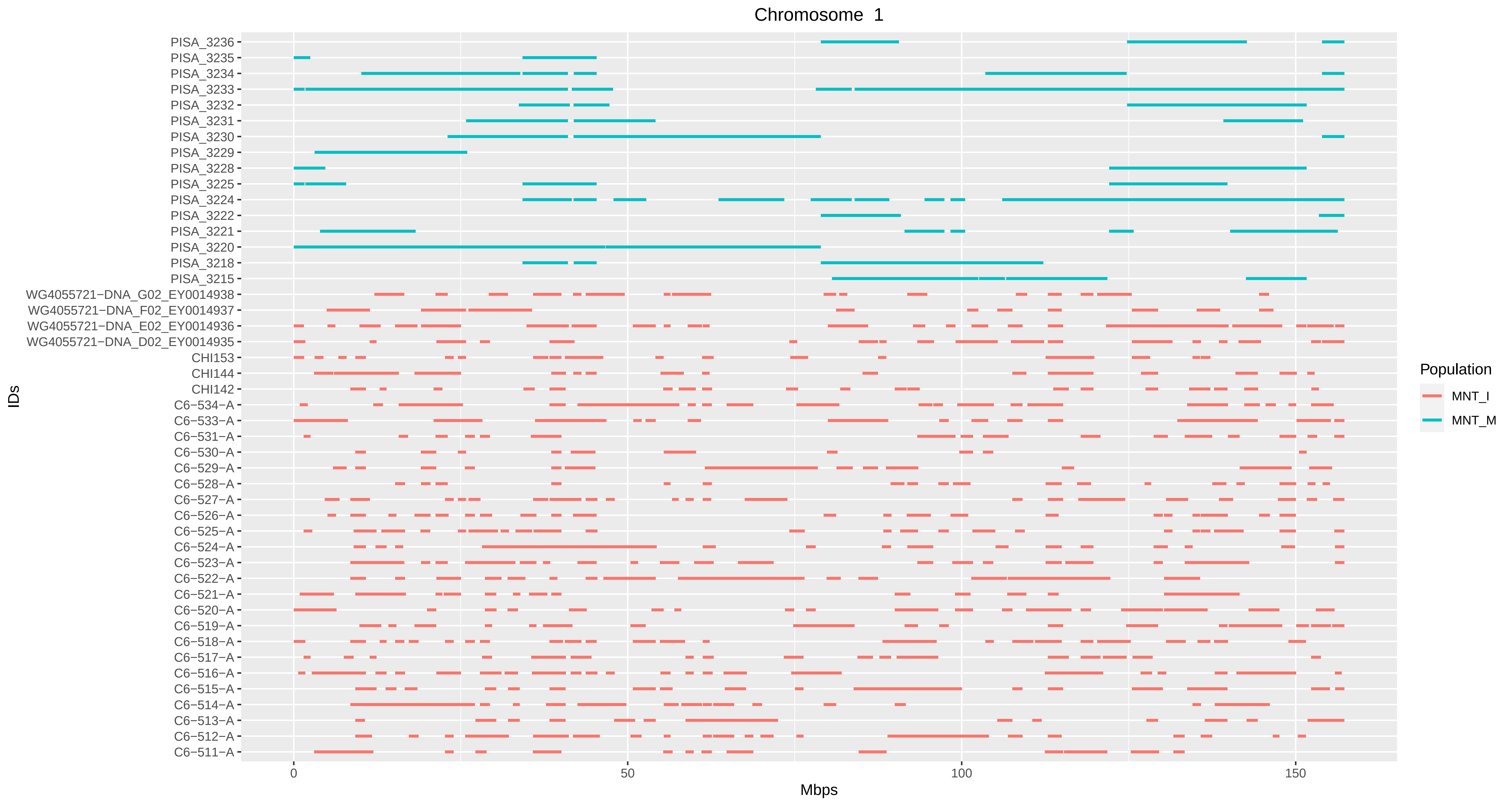

Chromosome 1
PISA_3236
PISA_3235
PISA_3234
PISA_3233
PISA_3232
PISA_3231
PISA_3230
PISA_3229
PISA_3228
PISA_3225
PISA_3224
PISA_3222
PISA_3221
PISA_3220
PISA_3218
PISA_3215
WG4055721−DNA_G02_EY0014938
WG4055721−DNA_F02_EY0014937
WG4055721−DNA_E02_EY0014936
WG4055721−DNA_D02_EY0014935
CHI153
CHI144
CHI142
C6−534−A
C6−533−A
C6−531−A
C6−530−A
C6−529−A
C6−528−A
C6−527−A
C6−526−A
C6−525−A
C6−524−A
C6−523−A
C6−522−A
C6−521−A
C6−520−A
C6−519−A
C6−518−A
C6−517−A
C6−516−A
C6−515−A
C6−514−A
C6−513−A
C6−512−A
C6−511−A
IDs
0
50
100
150
Mbps
Population
MNT_I
MNT_M

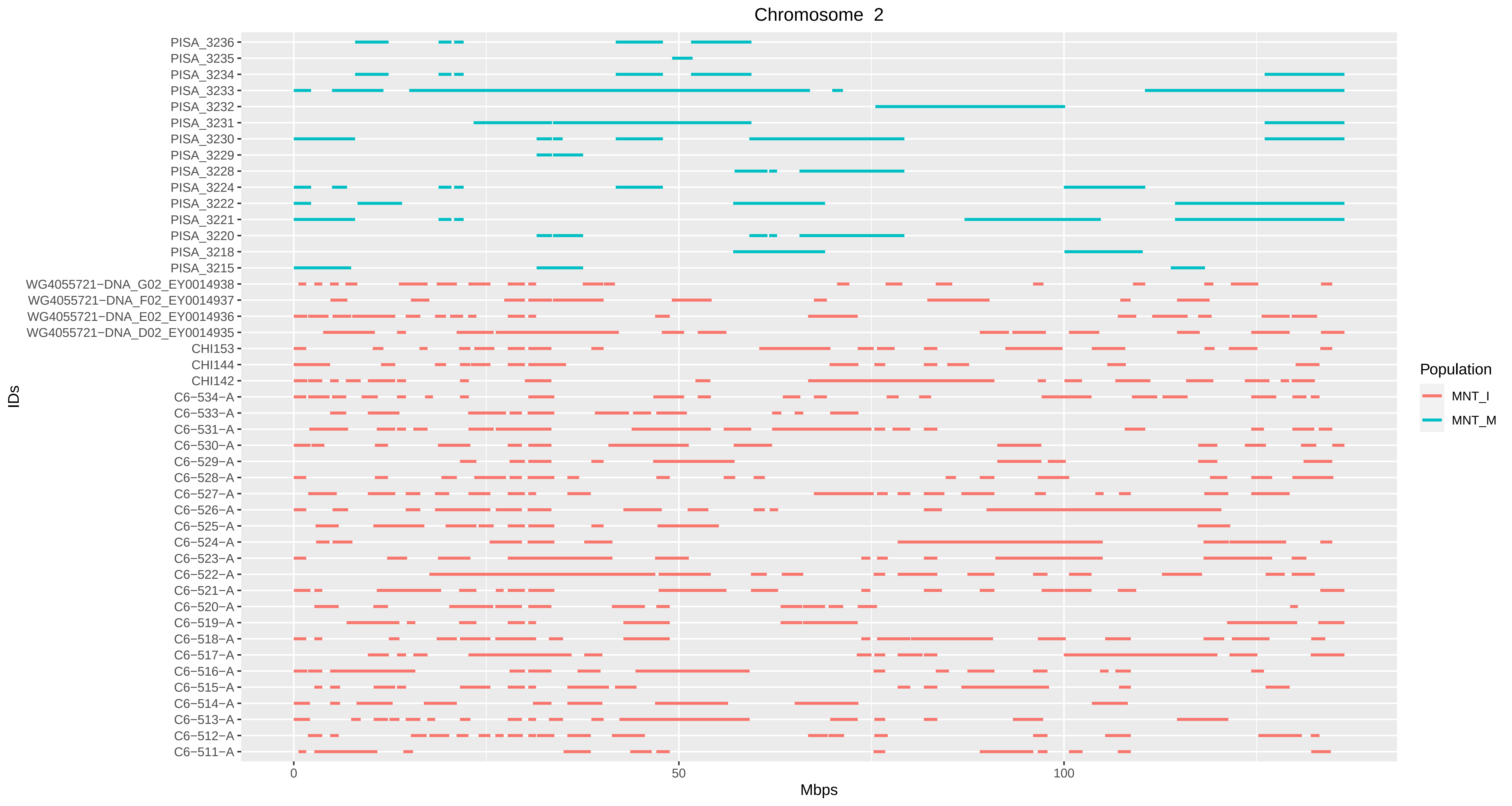

Chromosome 2
PISA_3236
PISA_3235
PISA_3234
PISA_3233
PISA_3232
PISA_3231
PISA_3230
PISA_3229
PISA_3228
PISA_3224
PISA_3222
PISA_3221
PISA_3220
PISA_3218
PISA_3215
WG4055721−DNA_G02_EY0014938
WG4055721−DNA_F02_EY0014937
WG4055721−DNA_E02_EY0014936
WG4055721−DNA_D02_EY0014935
CHI153
CHI144
CHI142
C6−534−A
C6−533−A
C6−531−A
C6−530−A
C6−529−A
C6−528−A
C6−527−A
C6−526−A
C6−525−A
C6−524−A
C6−523−A
C6−522−A
C6−521−A
C6−520−A
C6−519−A
C6−518−A
C6−517−A
C6−516−A
C6−515−A
C6−514−A
C6−513−A
C6−512−A
C6−511−A
IDs
0
50
100
Mbps
Population
MNT_I
MNT_M

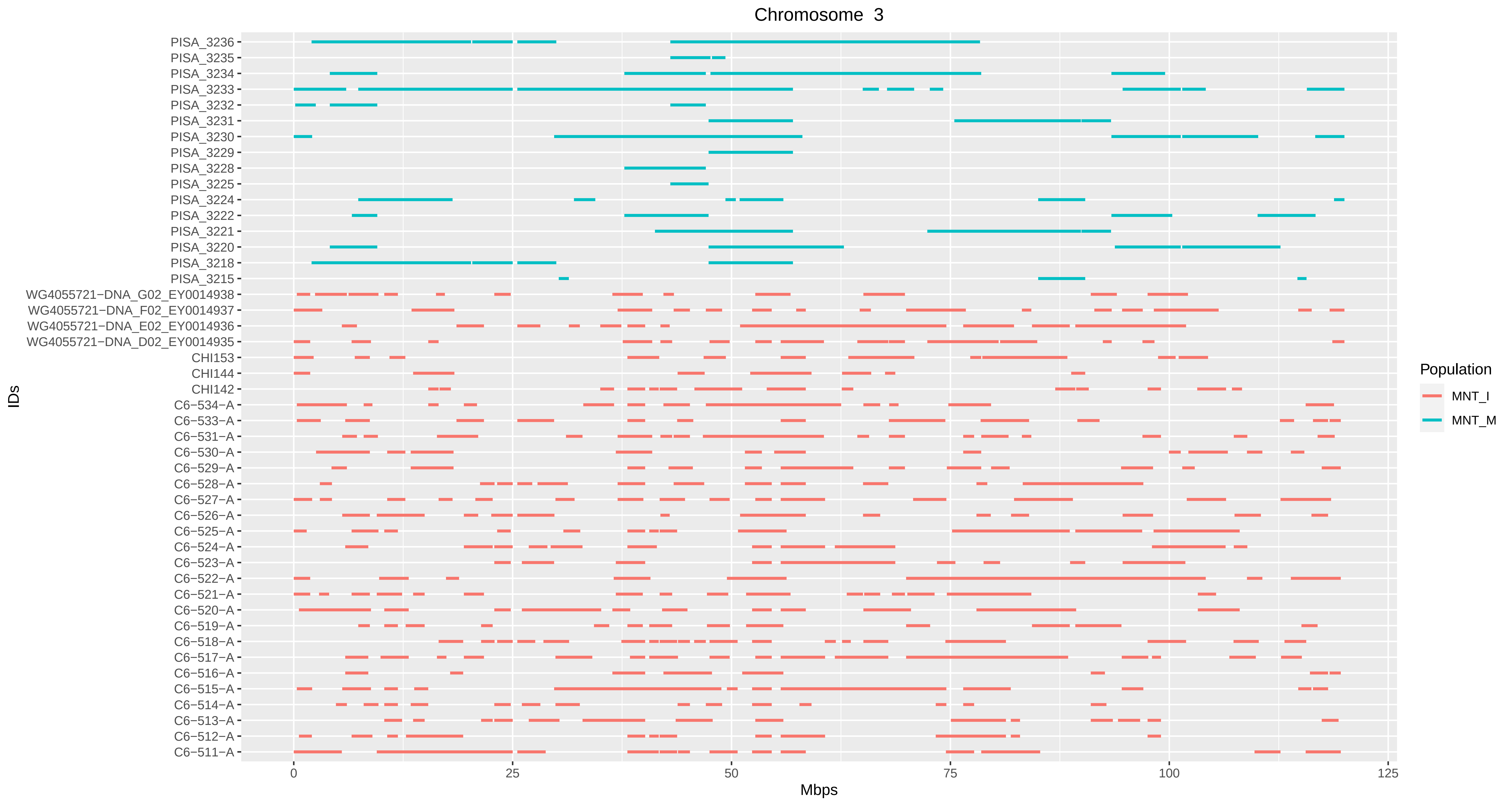
Chromosome 3
PISA_3236
PISA_3235
PISA_3234
PISA_3233
PISA_3232
PISA_3231
PISA_3230
PISA_3229
PISA_3228
PISA_3225
PISA_3224
PISA_3222
PISA_3221
PISA_3220
PISA_3218
PISA_3215
WG4055721−DNA_G02_EY0014938
WG4055721−DNA_F02_EY0014937
WG4055721−DNA_E02_EY0014936
WG4055721−DNA_D02_EY0014935
CHI153
CHI144
CHI142
C6−534−A
C6−533−A
C6−531−A
C6−530−A
C6−529−A
C6−528−A
C6−527−A
C6−526−A
C6−525−A
C6−524−A
C6−523−A
C6−522−A
C6−521−A
C6−520−A
C6−519−A
C6−518−A
C6−517−A
C6−516−A
C6−515−A
C6−514−A
C6−513−A
C6−512−A
C6−511−A
IDs
0
25
50
75
100
125
Mbps
Population
MNT_I
MNT_M

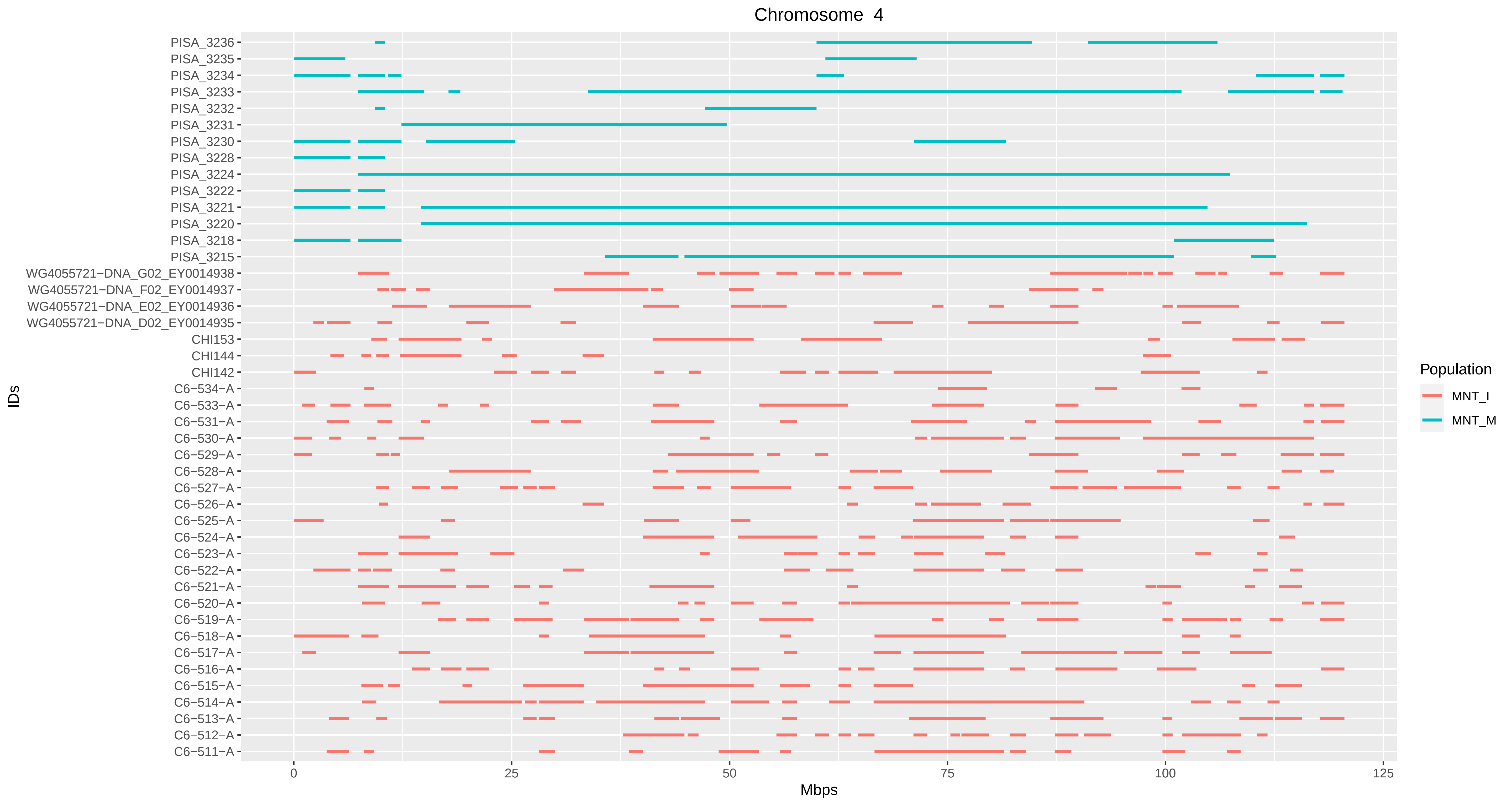
Chromosome 4
PISA_3236
PISA_3235
PISA_3234
PISA_3233
PISA_3232
PISA_3231
PISA_3230
PISA_3228
PISA_3224
PISA_3222
PISA_3221
PISA_3220
PISA_3218
PISA_3215
WG4055721−DNA_G02_EY0014938
WG4055721−DNA_F02_EY0014937
WG4055721−DNA_E02_EY0014936
WG4055721−DNA_D02_EY0014935
CHI153
CHI144
CHI142
C6−534−A
C6−533−A
C6−531−A
C6−530−A
C6−529−A
C6−528−A
C6−527−A
C6−526−A
C6−525−A
C6−524−A
C6−523−A
C6−522−A
C6−521−A
C6−520−A
C6−519−A
C6−518−A
C6−517−A
C6−516−A
C6−515−A
C6−514−A
C6−513−A
C6−512−A
C6−511−A
IDs
0
25
50
75
100
125
Mbps
Population
MNT_I
MNT_M

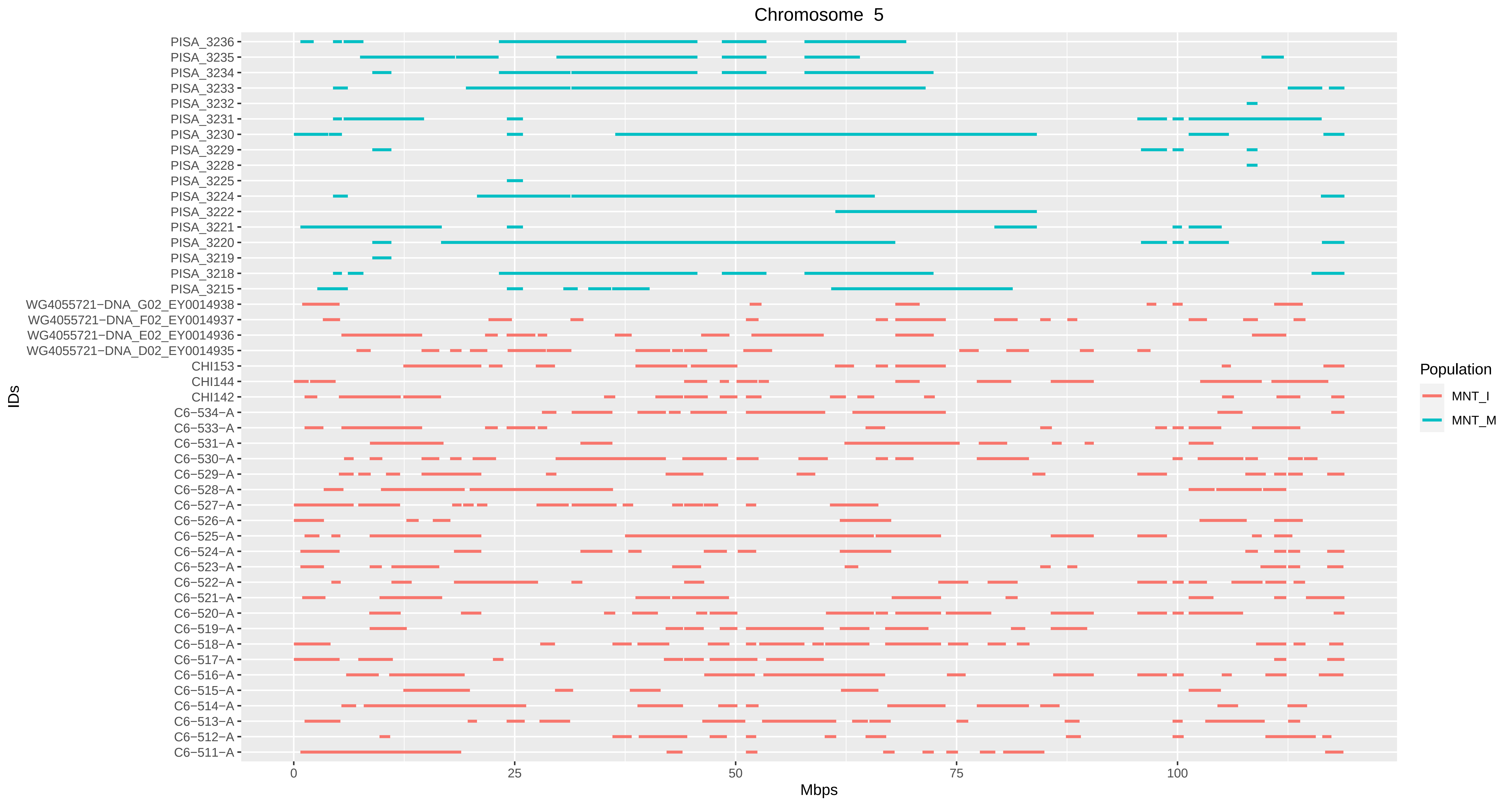

Chromosome 5
PISA_3236
PISA_3235
PISA_3234
PISA_3233
PISA_3232
PISA_3231
PISA_3230
PISA_3229
PISA_3228
PISA_3225
PISA_3224
PISA_3222
PISA_3221
PISA_3220
PISA_3219
PISA_3218
PISA_3215
WG4055721−DNA_G02_EY0014938
WG4055721−DNA_F02_EY0014937
WG4055721−DNA_E02_EY0014936
WG4055721−DNA_D02_EY0014935
CHI153
CHI144
CHI142
C6−534−A
C6−533−A
C6−531−A
C6−530−A
C6−529−A
C6−528−A
C6−527−A
C6−526−A
C6−525−A
C6−524−A
C6−523−A
C6−522−A
C6−521−A
C6−520−A
C6−519−A
C6−518−A
C6−517−A
C6−516−A
C6−515−A
C6−514−A
C6−513−A
C6−512−A
C6−511−A
IDs
0
25
50
75
100
Mbps
Population
MNT_I
MNT_M

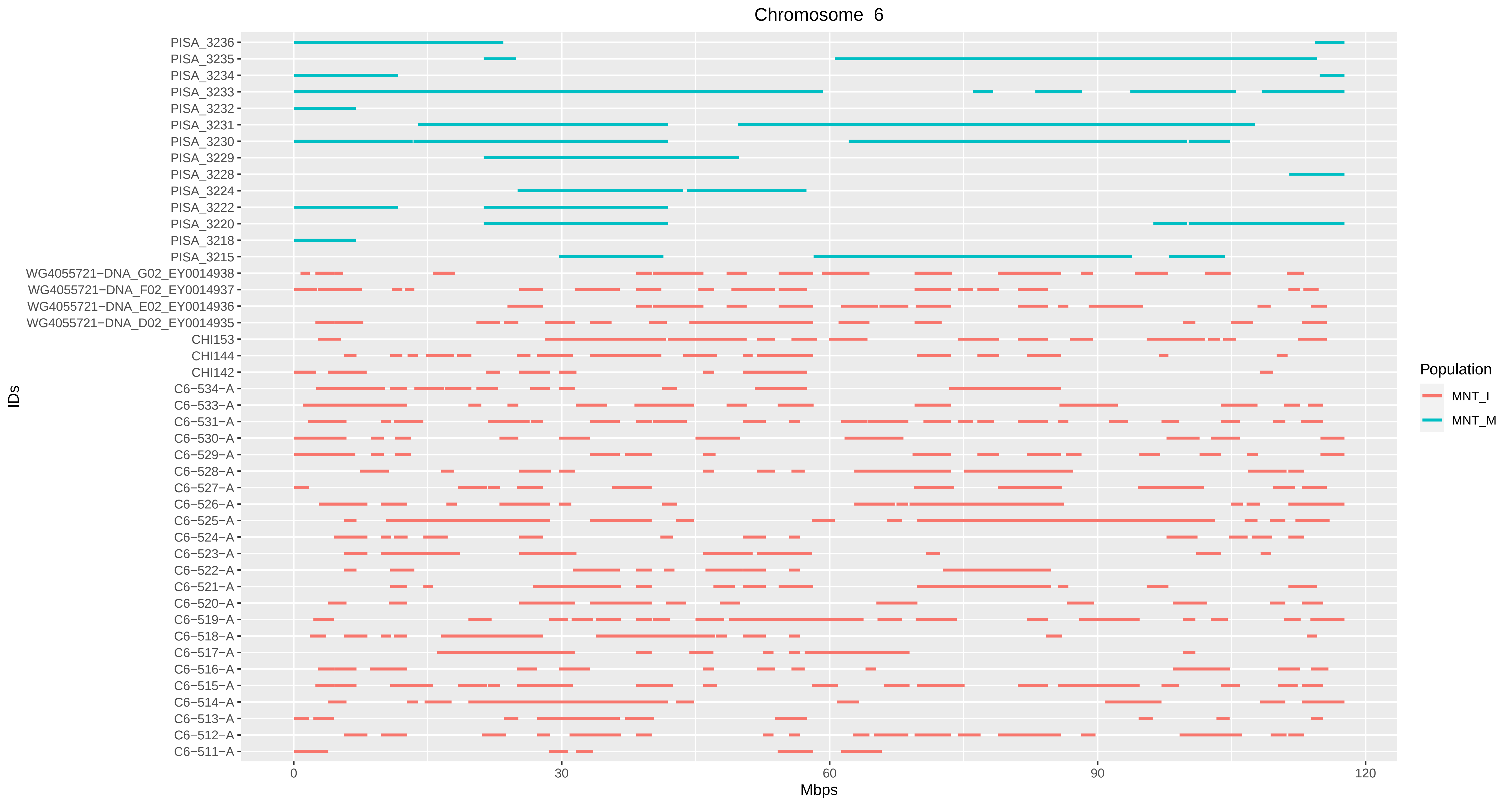

Chromosome 6
PISA_3236
PISA_3235
PISA_3234
PISA_3233
PISA_3232
PISA_3231
PISA_3230
PISA_3229
PISA_3228
PISA_3224
PISA_3222
PISA_3220
PISA_3218
PISA_3215
WG4055721−DNA_G02_EY0014938
WG4055721−DNA_F02_EY0014937
WG4055721−DNA_E02_EY0014936
WG4055721−DNA_D02_EY0014935
CHI153
CHI144
CHI142
C6−534−A
C6−533−A
C6−531−A
C6−530−A
C6−529−A
C6−528−A
C6−527−A
C6−526−A
C6−525−A
C6−524−A
C6−523−A
C6−522−A
C6−521−A
C6−520−A
C6−519−A
C6−518−A
C6−517−A
C6−516−A
C6−515−A
C6−514−A
C6−513−A
C6−512−A
C6−511−A
IDs
0
30
60
90
120
Mbps
Population
MNT_I
MNT_M

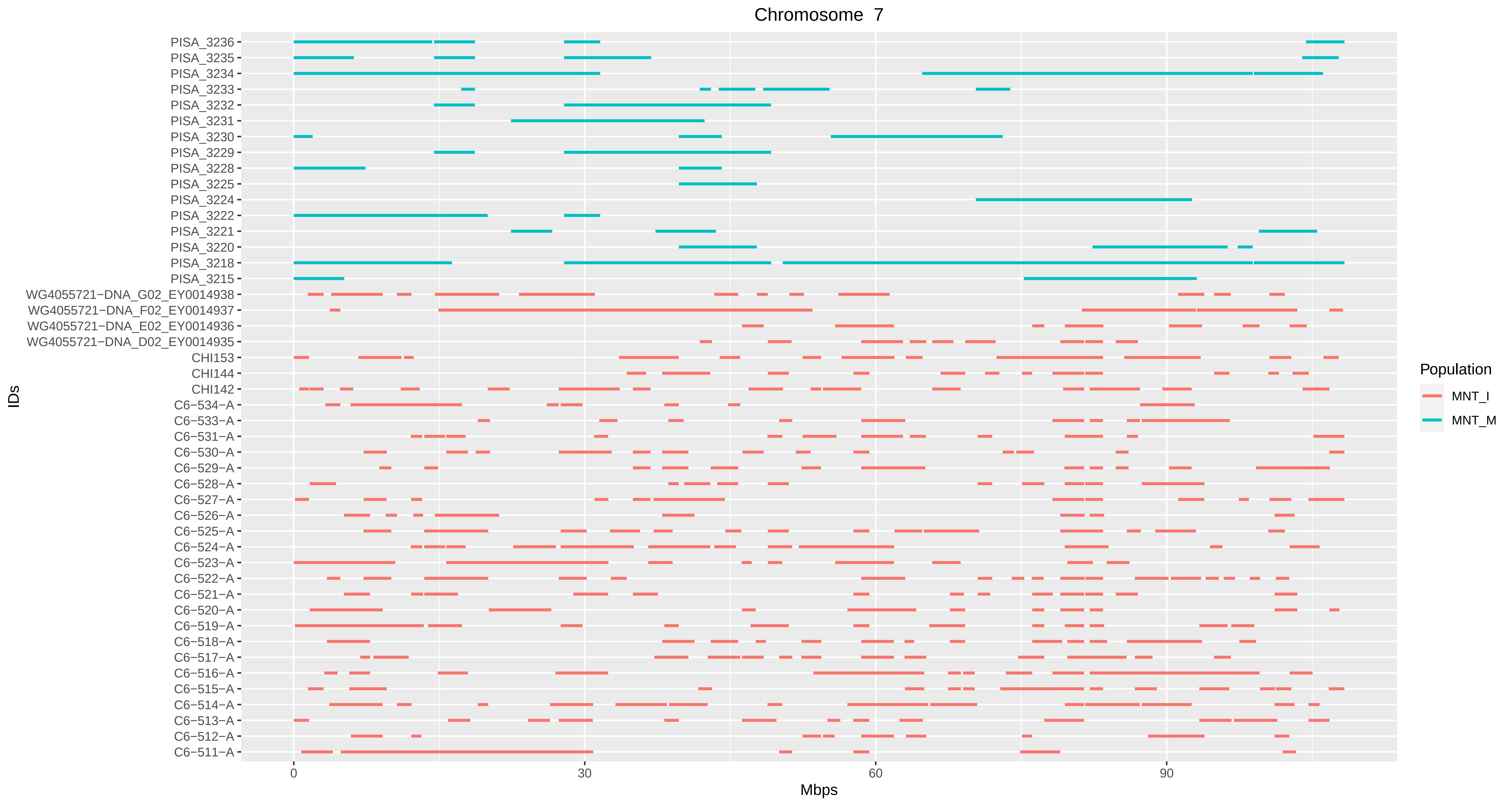

Chromosome 7
PISA_3236
PISA_3235
PISA_3234
PISA_3233
PISA_3232
PISA_3231
PISA_3230
PISA_3229
PISA_3228
PISA_3225
PISA_3224
PISA_3222
PISA_3221
PISA_3220
PISA_3218
PISA_3215
WG4055721−DNA_G02_EY0014938
WG4055721−DNA_F02_EY0014937
WG4055721−DNA_E02_EY0014936
WG4055721−DNA_D02_EY0014935
CHI153
CHI144
CHI142
C6−534−A
C6−533−A
C6−531−A
C6−530−A
C6−529−A
C6−528−A
C6−527−A
C6−526−A
C6−525−A
C6−524−A
C6−523−A
C6−522−A
C6−521−A
C6−520−A
C6−519−A
C6−518−A
C6−517−A
C6−516−A
C6−515−A
C6−514−A
C6−513−A
C6−512−A
C6−511−A
IDs
0
30
60
90
Mbps
Population
MNT_I
MNT_M

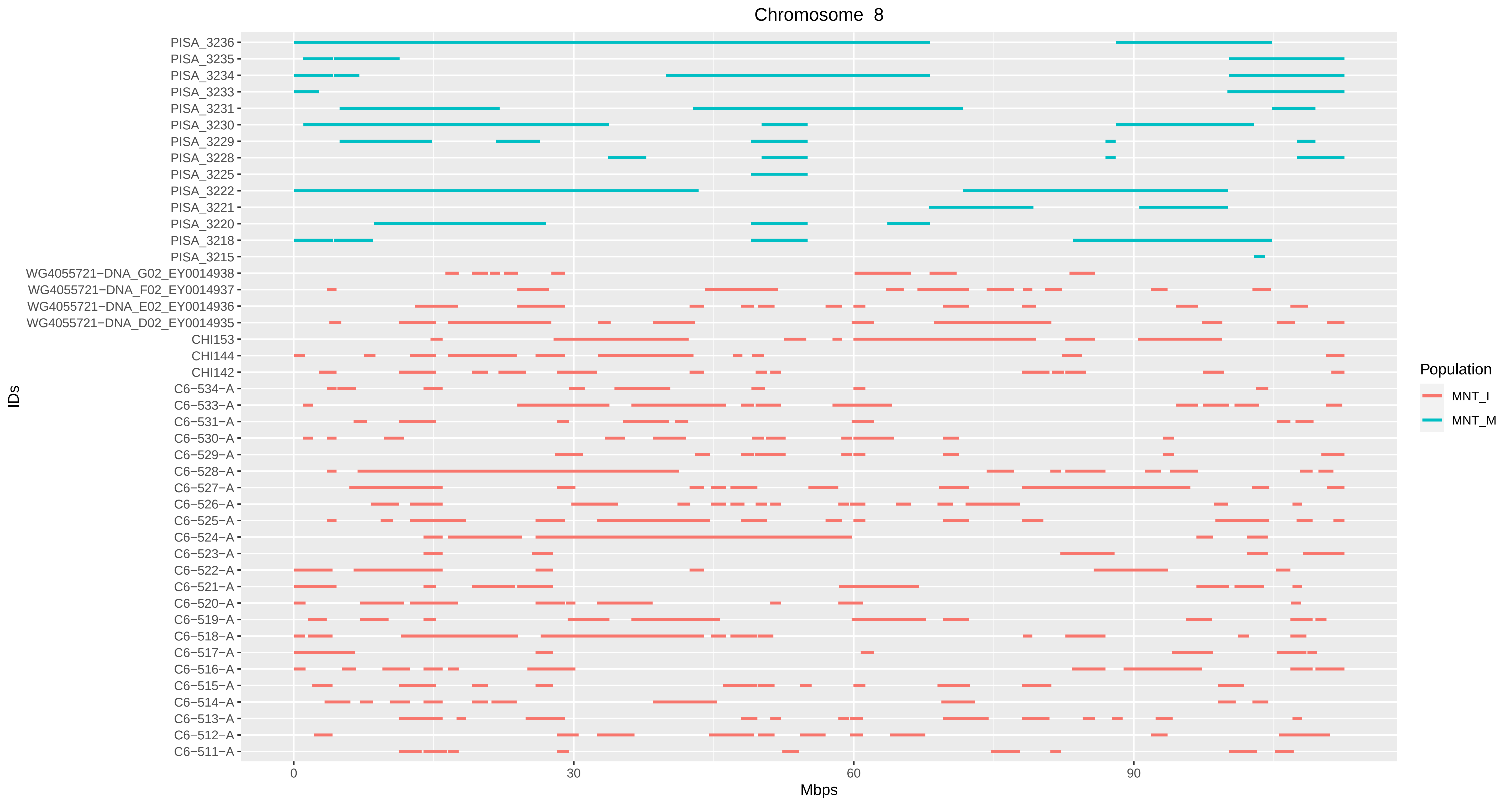

Chromosome 8
PISA_3236
PISA_3235
PISA_3234
PISA_3233
PISA_3231
PISA_3230
PISA_3229
PISA_3228
PISA_3225
PISA_3222
PISA_3221
PISA_3220
PISA_3218
PISA_3215
WG4055721−DNA_G02_EY0014938
WG4055721−DNA_F02_EY0014937
WG4055721−DNA_E02_EY0014936
WG4055721−DNA_D02_EY0014935
CHI153
CHI144
CHI142
C6−534−A
C6−533−A
C6−531−A
C6−530−A
C6−529−A
C6−528−A
C6−527−A
C6−526−A
C6−525−A
C6−524−A
C6−523−A
C6−522−A
C6−521−A
C6−520−A
C6−519−A
C6−518−A
C6−517−A
C6−516−A
C6−515−A
C6−514−A
C6−513−A
C6−512−A
C6−511−A
IDs
0
30
60
90
Mbps
Population
MNT_I
MNT_M

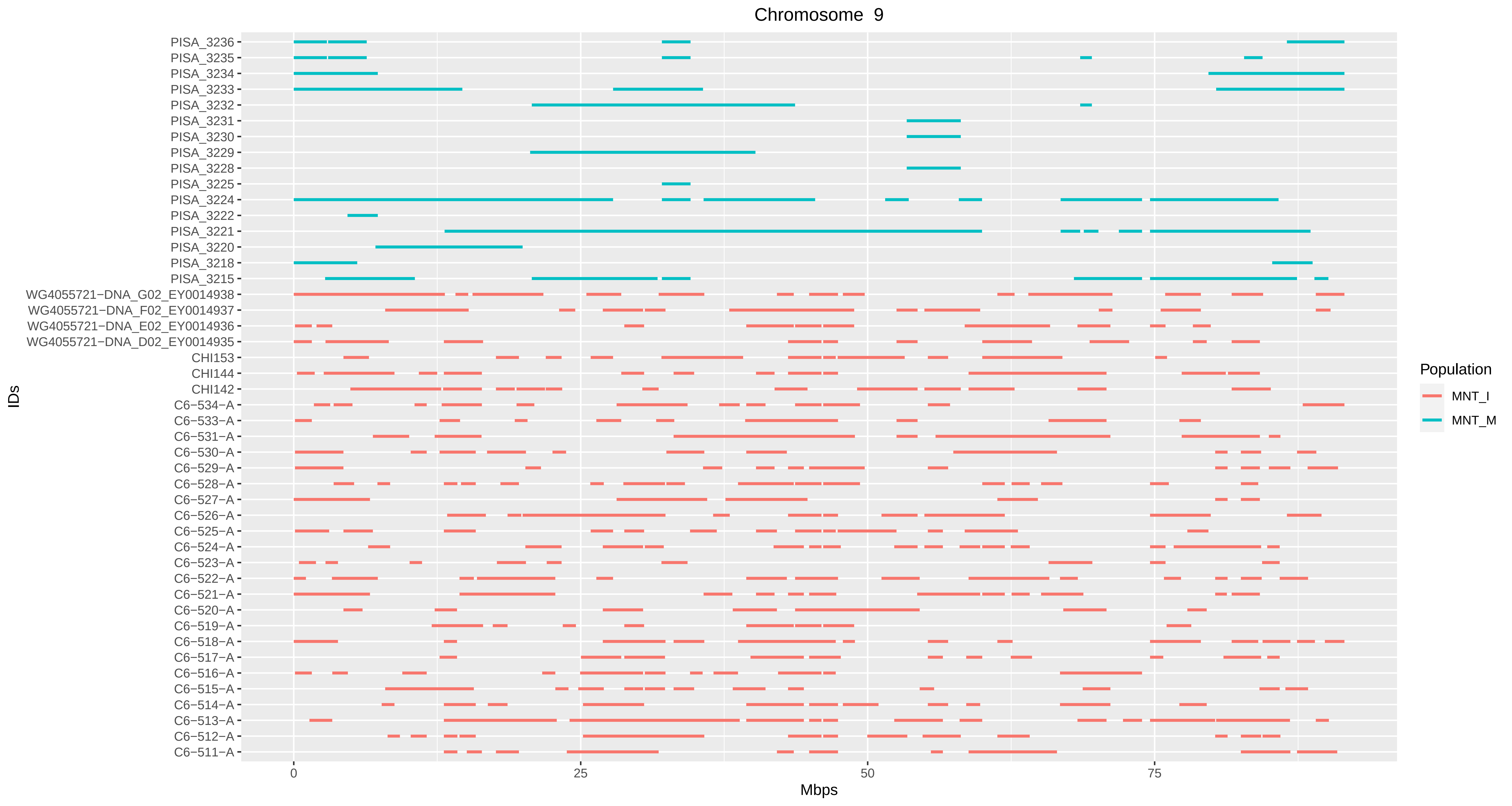

Chromosome 9
PISA_3236
PISA_3235
PISA_3234
PISA_3233
PISA_3232
PISA_3231
PISA_3230
PISA_3229
PISA_3228
PISA_3225
PISA_3224
PISA_3222
PISA_3221
PISA_3220
PISA_3218
PISA_3215
WG4055721−DNA_G02_EY0014938
WG4055721−DNA_F02_EY0014937
WG4055721−DNA_E02_EY0014936
WG4055721−DNA_D02_EY0014935
CHI153
CHI144
CHI142
C6−534−A
C6−533−A
C6−531−A
C6−530−A
C6−529−A
C6−528−A
C6−527−A
C6−526−A
C6−525−A
C6−524−A
C6−523−A
C6−522−A
C6−521−A
C6−520−A
C6−519−A
C6−518−A
C6−517−A
C6−516−A
C6−515−A
C6−514−A
C6−513−A
C6−512−A
C6−511−A
IDs
0
25
50
75
Mbps
Population
MNT_I
MNT_M

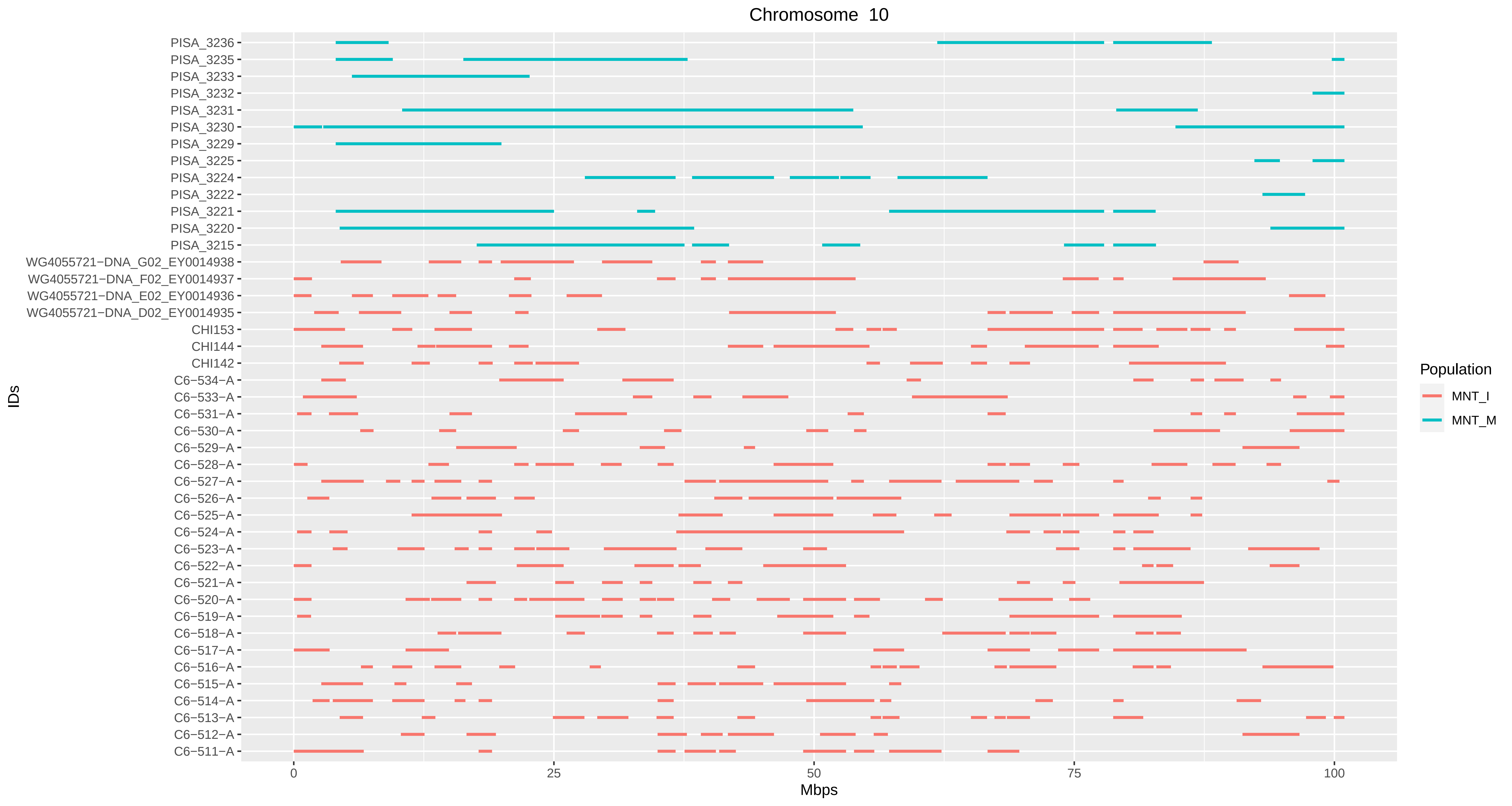

Chromosome 10
PISA_3236
PISA_3235
PISA_3233
PISA_3232
PISA_3231
PISA_3230
PISA_3229
PISA_3225
PISA_3224
PISA_3222
PISA_3221
PISA_3220
PISA_3215
WG4055721−DNA_G02_EY0014938
WG4055721−DNA_F02_EY0014937
WG4055721−DNA_E02_EY0014936
WG4055721−DNA_D02_EY0014935
CHI153
CHI144
CHI142
C6−534−A
C6−533−A
C6−531−A
C6−530−A
C6−529−A
C6−528−A
C6−527−A
C6−526−A
C6−525−A
C6−524−A
C6−523−A
C6−522−A
C6−521−A
C6−520−A
C6−519−A
C6−518−A
C6−517−A
C6−516−A
C6−515−A
C6−514−A
C6−513−A
C6−512−A
C6−511−A
IDs
0
25
50
75
100
Mbps
Population
MNT_I
MNT_M

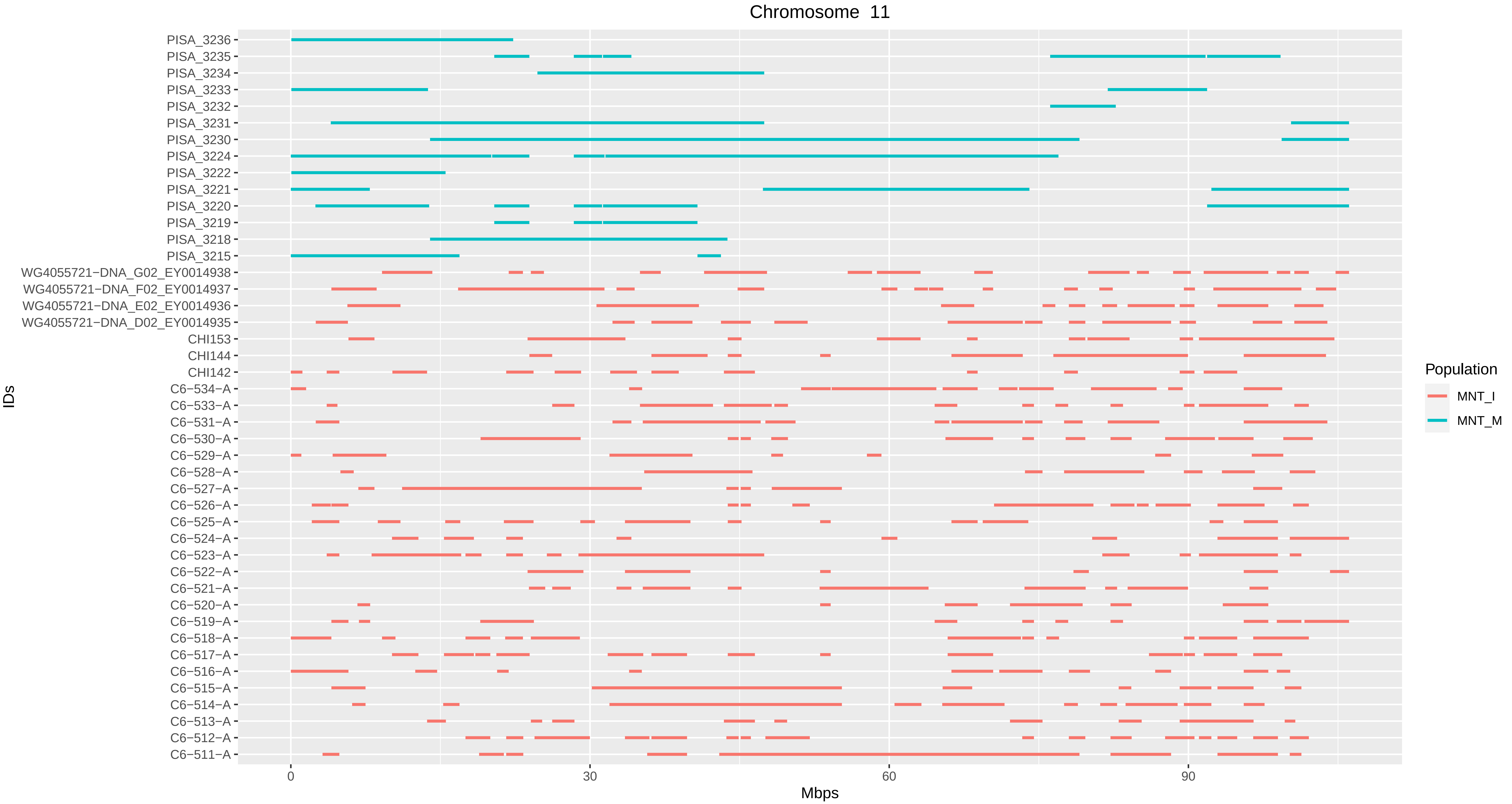

Chromosome 11
PISA_3236
PISA_3235
PISA_3234
PISA_3233
PISA_3232
PISA_3231
PISA_3230
PISA_3224
PISA_3222
PISA_3221
PISA_3220
PISA_3219
PISA_3218
PISA_3215
WG4055721−DNA_G02_EY0014938
WG4055721−DNA_F02_EY0014937
WG4055721−DNA_E02_EY0014936
WG4055721−DNA_D02_EY0014935
CHI153
CHI144
CHI142
C6−534−A
C6−533−A
C6−531−A
C6−530−A
C6−529−A
C6−528−A
C6−527−A
C6−526−A
C6−525−A
C6−524−A
C6−523−A
C6−522−A
C6−521−A
C6−520−A
C6−519−A
C6−518−A
C6−517−A
C6−516−A
C6−515−A
C6−514−A
C6−513−A
C6−512−A
C6−511−A
IDs
0
30
60
90
Mbps
Population
MNT_I
MNT_M

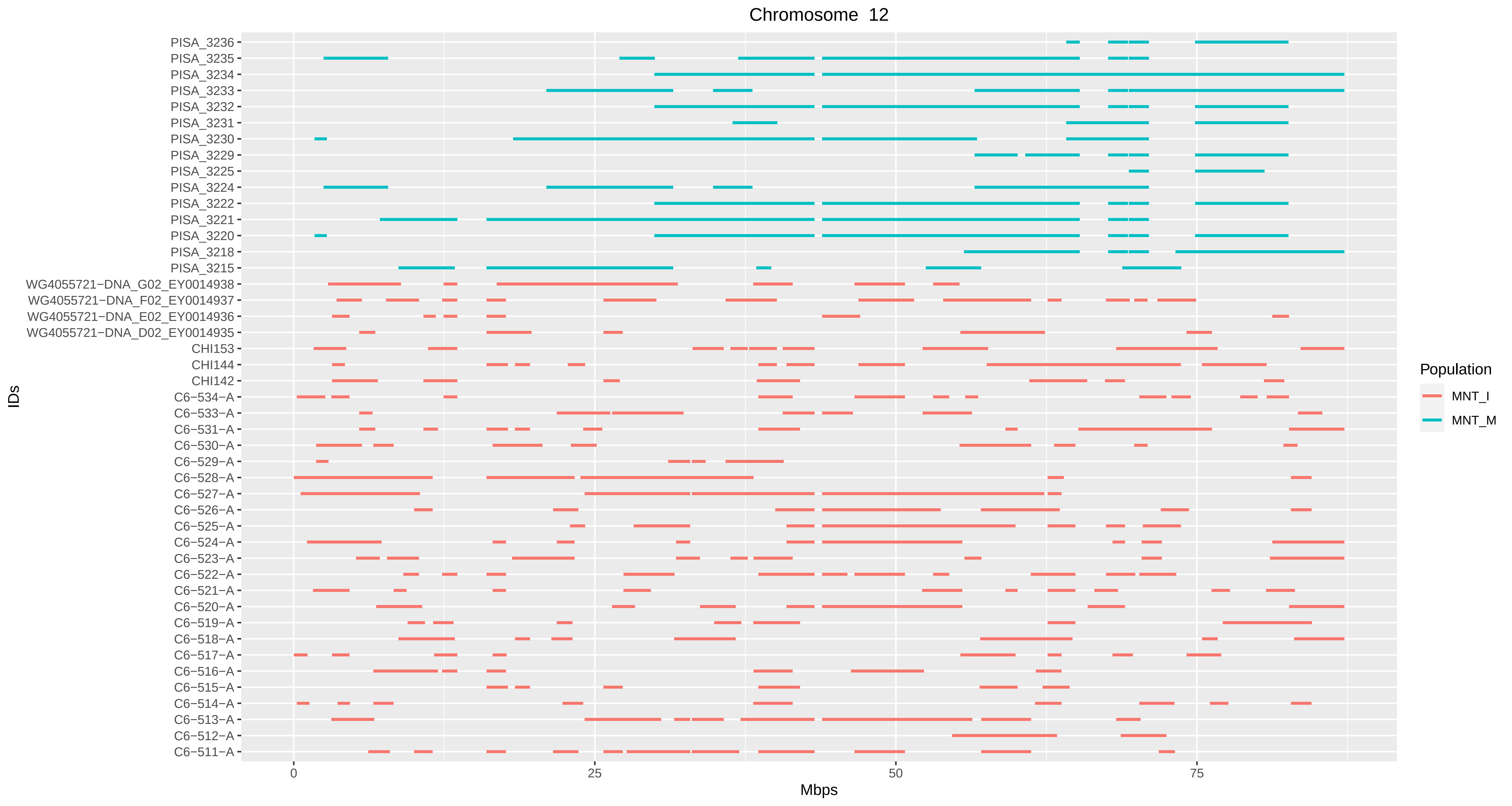

Chromosome 12
PISA_3236
PISA_3235
PISA_3234
PISA_3233
PISA_3232
PISA_3231
PISA_3230
PISA_3229
PISA_3225
PISA_3224
PISA_3222
PISA_3221
PISA_3220
PISA_3218
PISA_3215
WG4055721−DNA_G02_EY0014938
WG4055721−DNA_F02_EY0014937
WG4055721−DNA_E02_EY0014936
WG4055721−DNA_D02_EY0014935
CHI153
CHI144
CHI142
C6−534−A
C6−533−A
C6−531−A
C6−530−A
C6−529−A
C6−528−A
C6−527−A
C6−526−A
C6−525−A
C6−524−A
C6−523−A
C6−522−A
C6−521−A
C6−520−A
C6−519−A
C6−518−A
C6−517−A
C6−516−A
C6−515−A
C6−514−A
C6−513−A
C6−512−A
C6−511−A
IDs
0
25
50
75
Mbps
Population
MNT_I
MNT_M

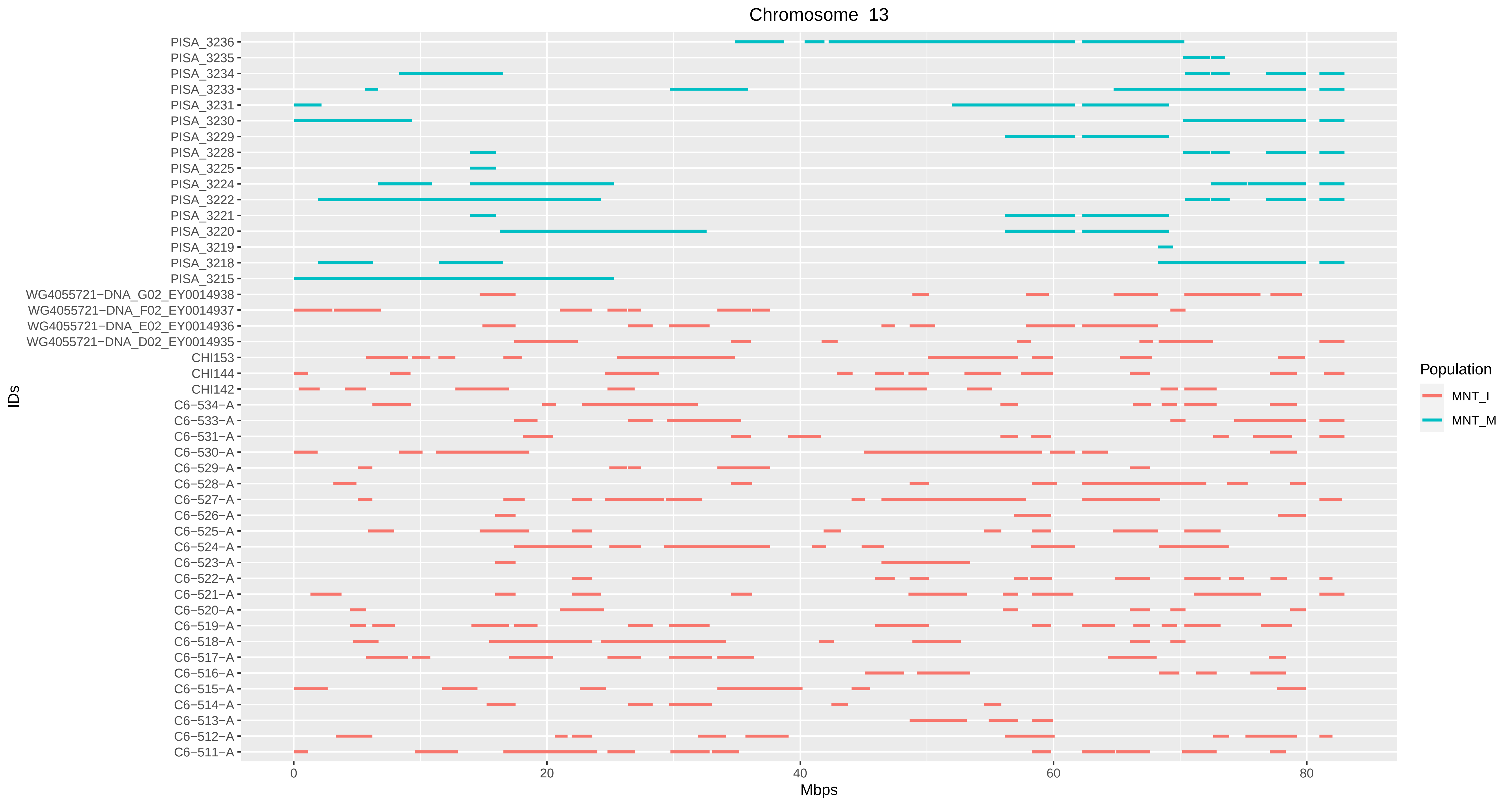
Chromosome 13
PISA_3236
PISA_3235
PISA_3234
PISA_3233
PISA_3231
PISA_3230
PISA_3229
PISA_3228
PISA_3225
PISA_3224
PISA_3222
PISA_3221
PISA_3220
PISA_3219
PISA_3218
PISA_3215
WG4055721−DNA_G02_EY0014938
WG4055721−DNA_F02_EY0014937
WG4055721−DNA_E02_EY0014936
WG4055721−DNA_D02_EY0014935
CHI153
CHI144
CHI142
C6−534−A
C6−533−A
C6−531−A
C6−530−A
C6−529−A
C6−528−A
C6−527−A
C6−526−A
C6−525−A
C6−524−A
C6−523−A
C6−522−A
C6−521−A
C6−520−A
C6−519−A
C6−518−A
C6−517−A
C6−516−A
C6−515−A
C6−514−A
C6−513−A
C6−512−A
C6−511−A
IDs
0
20
40
60
80
Mbps
Population
MNT_I
MNT_M

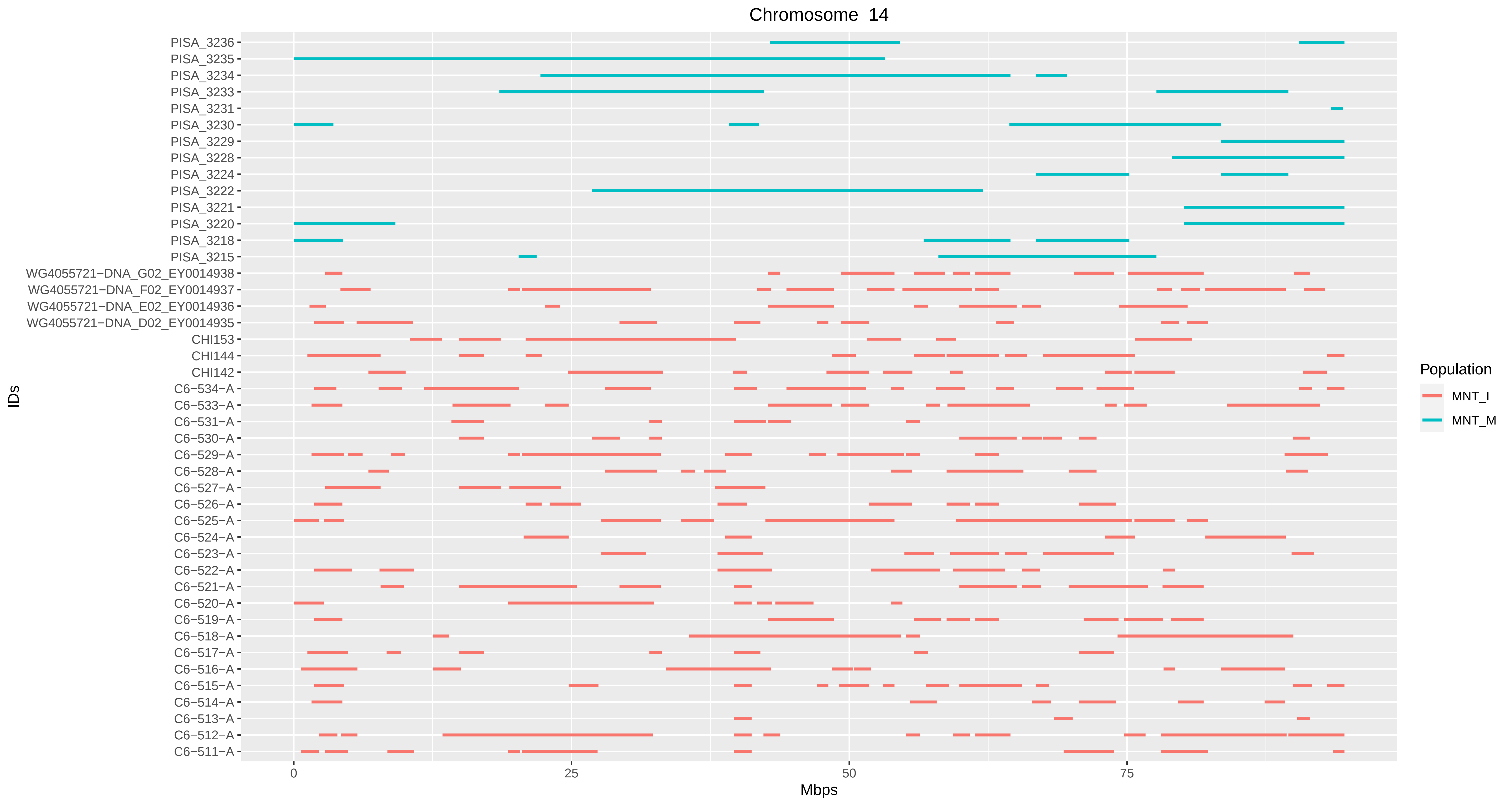

Chromosome 14
PISA_3236
PISA_3235
PISA_3234
PISA_3233
PISA_3231
PISA_3230
PISA_3229
PISA_3228
PISA_3224
PISA_3222
PISA_3221
PISA_3220
PISA_3218
PISA_3215
WG4055721−DNA_G02_EY0014938
WG4055721−DNA_F02_EY0014937
WG4055721−DNA_E02_EY0014936
WG4055721−DNA_D02_EY0014935
CHI153
CHI144
CHI142
C6−534−A
C6−533−A
C6−531−A
C6−530−A
C6−529−A
C6−528−A
C6−527−A
C6−526−A
C6−525−A
C6−524−A
C6−523−A
C6−522−A
C6−521−A
C6−520−A
C6−519−A
C6−518−A
C6−517−A
C6−516−A
C6−515−A
C6−514−A
C6−513−A
C6−512−A
C6−511−A
IDs
0
25
50
75
Mbps
Population
MNT_I
MNT_M

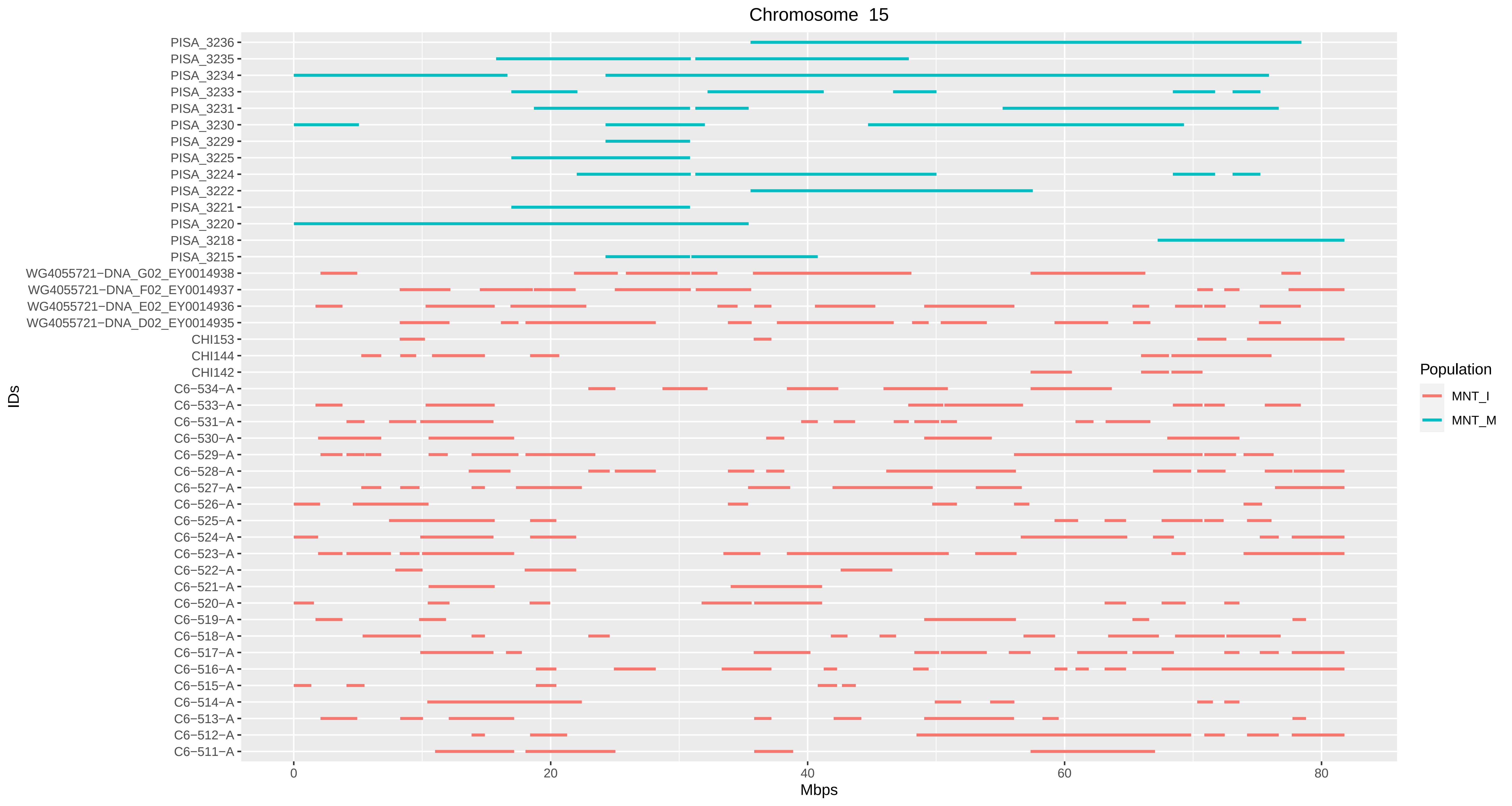
Chromosome 15
PISA_3236
PISA_3235
PISA_3234
PISA_3233
PISA_3231
PISA_3230
PISA_3229
PISA_3225
PISA_3224
PISA_3222
PISA_3221
PISA_3220
PISA_3218
PISA_3215
WG4055721−DNA_G02_EY0014938
WG4055721−DNA_F02_EY0014937
WG4055721−DNA_E02_EY0014936
WG4055721−DNA_D02_EY0014935
CHI153
CHI144
CHI142
C6−534−A
C6−533−A
C6−531−A
C6−530−A
C6−529−A
C6−528−A
C6−527−A
C6−526−A
C6−525−A
C6−524−A
C6−523−A
C6−522−A
C6−521−A
C6−520−A
C6−519−A
C6−518−A
C6−517−A
C6−516−A
C6−515−A
C6−514−A
C6−513−A
C6−512−A
C6−511−A
IDs
0
20
40
60
80
Mbps
Population
MNT_I
MNT_M

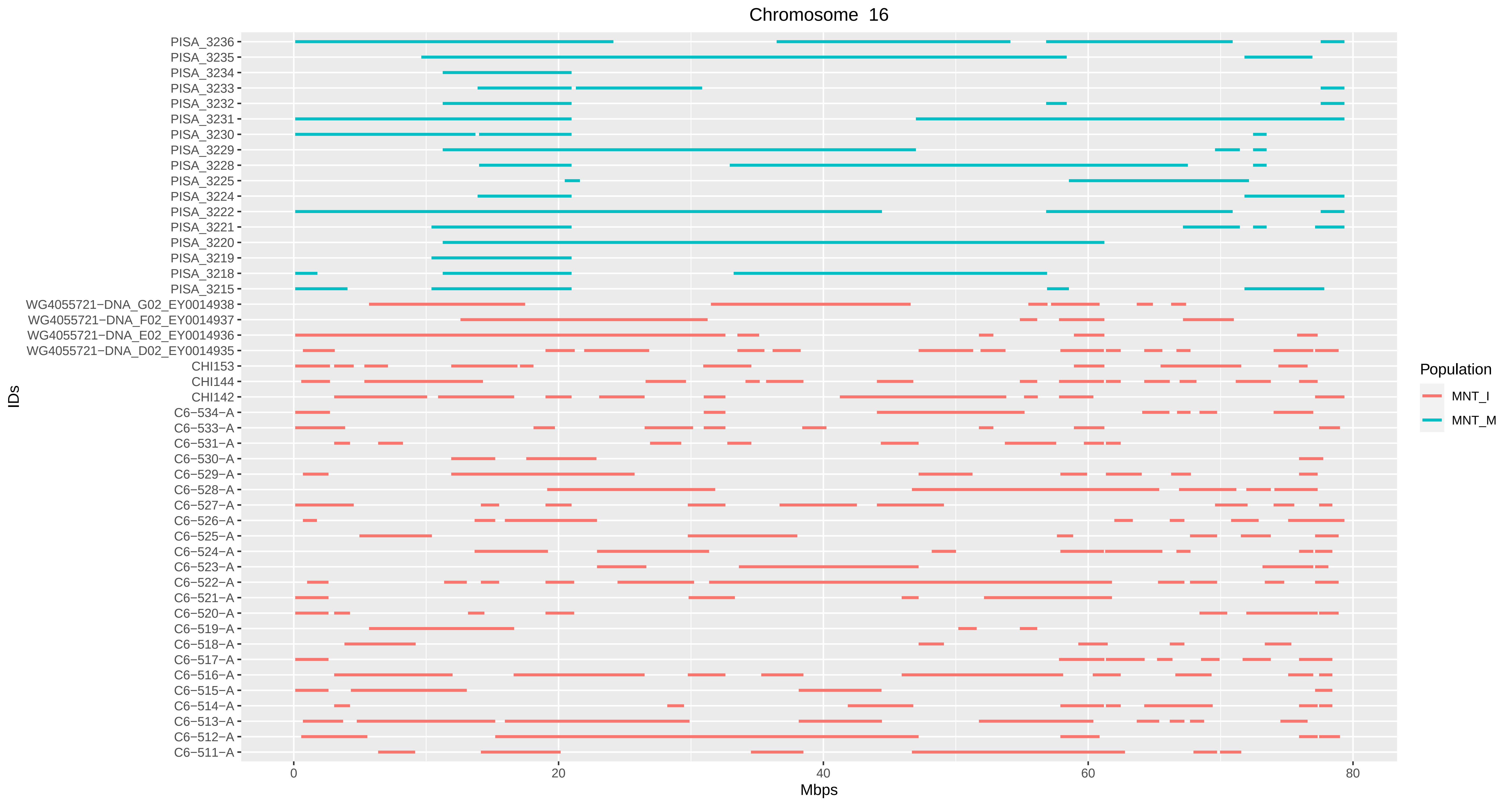
Chromosome 16
PISA_3236
PISA_3235
PISA_3234
PISA_3233
PISA_3232
PISA_3231
PISA_3230
PISA_3229
PISA_3228
PISA_3225
PISA_3224
PISA_3222
PISA_3221
PISA_3220
PISA_3219
PISA_3218
PISA_3215
WG4055721−DNA_G02_EY0014938
WG4055721−DNA_F02_EY0014937
WG4055721−DNA_E02_EY0014936
WG4055721−DNA_D02_EY0014935
CHI153
CHI144
CHI142
C6−534−A
C6−533−A
C6−531−A
C6−530−A
C6−529−A
C6−528−A
C6−527−A
C6−526−A
C6−525−A
C6−524−A
C6−523−A
C6−522−A
C6−521−A
C6−520−A
C6−519−A
C6−518−A
C6−517−A
C6−516−A
C6−515−A
C6−514−A
C6−513−A
C6−512−A
C6−511−A
IDs
0
20
40
60
80
Mbps
Population
MNT_I
MNT_M

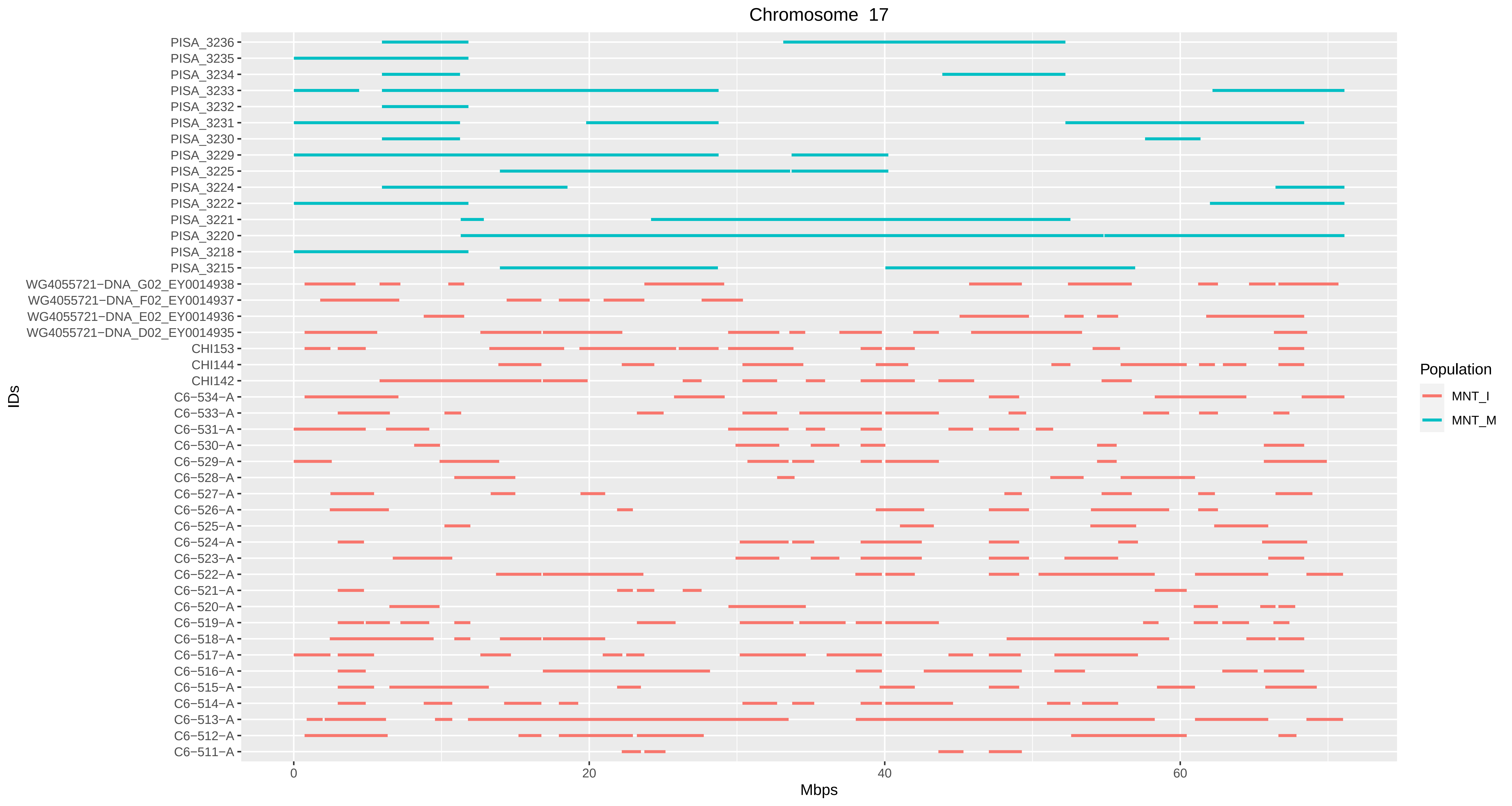

Chromosome 17
PISA_3236
PISA_3235
PISA_3234
PISA_3233
PISA_3232
PISA_3231
PISA_3230
PISA_3229
PISA_3225
PISA_3224
PISA_3222
PISA_3221
PISA_3220
PISA_3218
PISA_3215
WG4055721−DNA_G02_EY0014938
WG4055721−DNA_F02_EY0014937
WG4055721−DNA_E02_EY0014936
WG4055721−DNA_D02_EY0014935
CHI153
CHI144
CHI142
C6−534−A
C6−533−A
C6−531−A
C6−530−A
C6−529−A
C6−528−A
C6−527−A
C6−526−A
C6−525−A
C6−524−A
C6−523−A
C6−522−A
C6−521−A
C6−520−A
C6−519−A
C6−518−A
C6−517−A
C6−516−A
C6−515−A
C6−514−A
C6−513−A
C6−512−A
C6−511−A
IDs
0
20
40
60
Mbps
Population
MNT_I
MNT_M

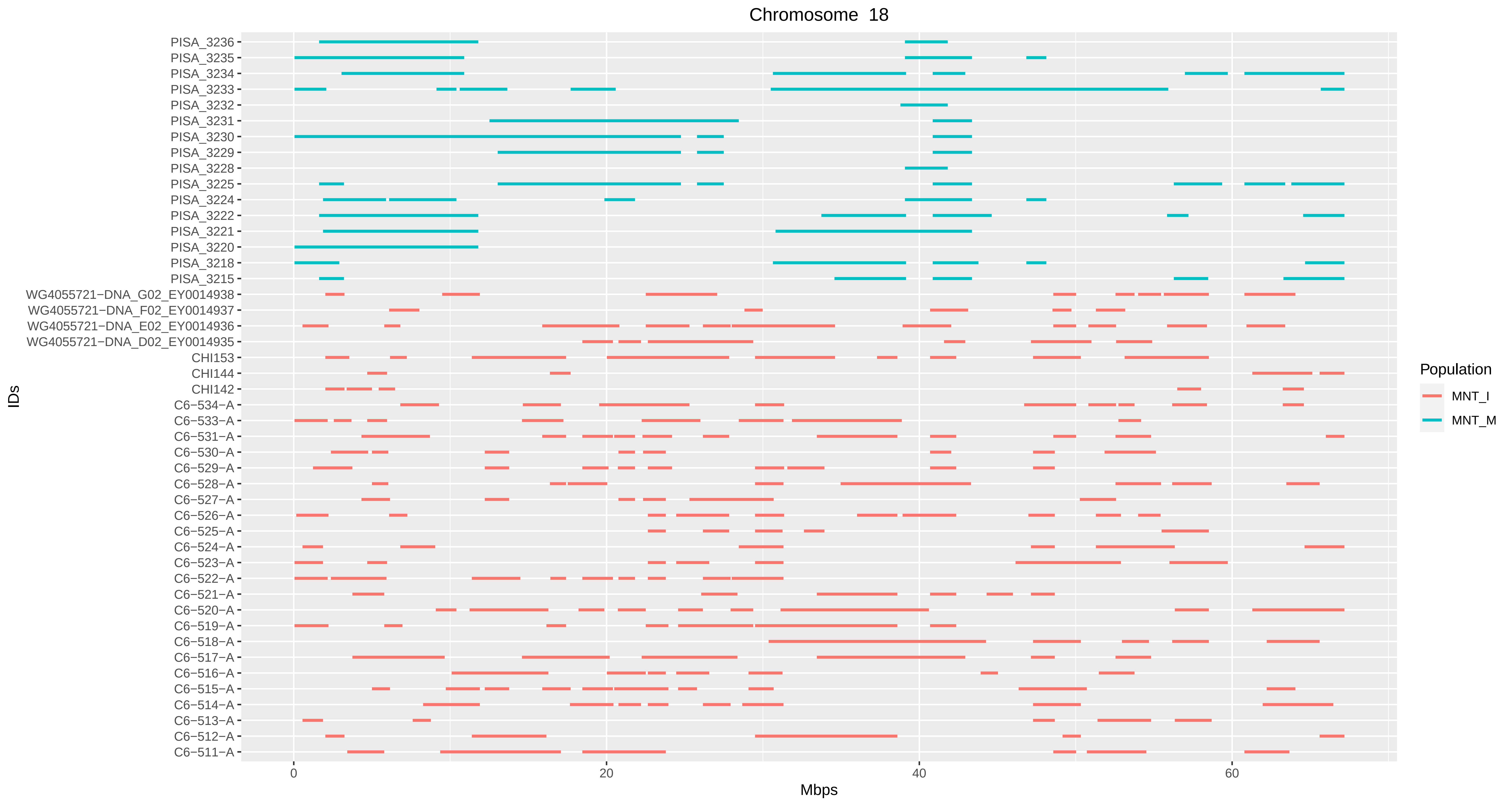

Chromosome 18
PISA_3236
PISA_3235
PISA_3234
PISA_3233
PISA_3232
PISA_3231
PISA_3230
PISA_3229
PISA_3228
PISA_3225
PISA_3224
PISA_3222
PISA_3221
PISA_3220
PISA_3218
PISA_3215
WG4055721−DNA_G02_EY0014938
WG4055721−DNA_F02_EY0014937
WG4055721−DNA_E02_EY0014936
WG4055721−DNA_D02_EY0014935
CHI153
CHI144
CHI142
C6−534−A
C6−533−A
C6−531−A
C6−530−A
C6−529−A
C6−528−A
C6−527−A
C6−526−A
C6−525−A
C6−524−A
C6−523−A
C6−522−A
C6−521−A
C6−520−A
C6−519−A
C6−518−A
C6−517−A
C6−516−A
C6−515−A
C6−514−A
C6−513−A
C6−512−A
C6−511−A
IDs
0
20
40
60
Mbps
Population
MNT_I
MNT_M

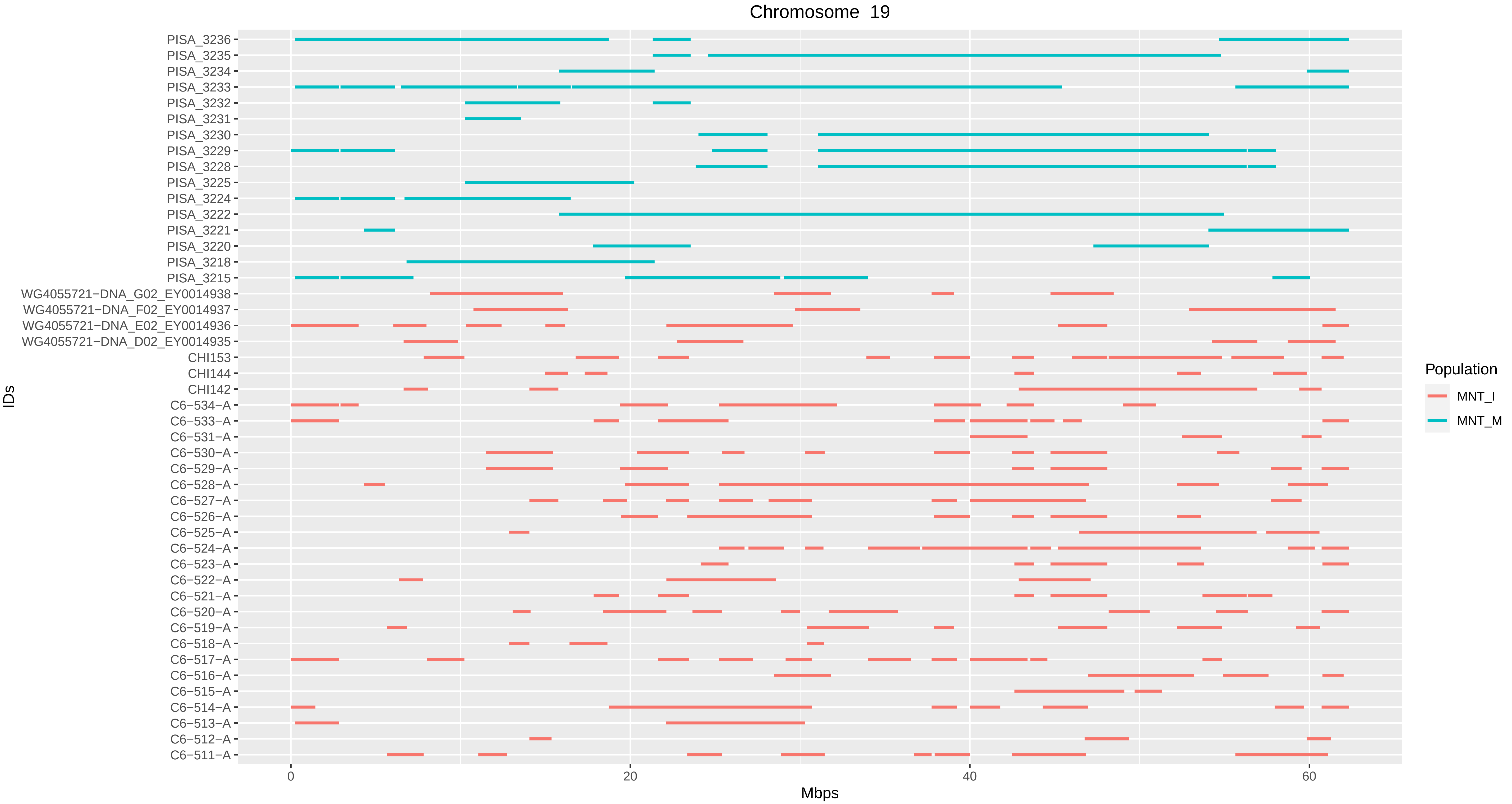
Chromosome 19
PISA_3236
PISA_3235
PISA_3234
PISA_3233
PISA_3232
PISA_3231
PISA_3230
PISA_3229
PISA_3228
PISA_3225
PISA_3224
PISA_3222
PISA_3221
PISA_3220
PISA_3218
PISA_3215
WG4055721−DNA_G02_EY0014938
WG4055721−DNA_F02_EY0014937
WG4055721−DNA_E02_EY0014936
WG4055721−DNA_D02_EY0014935
CHI153
CHI144
CHI142
C6−534−A
C6−533−A
C6−531−A
C6−530−A
C6−529−A
C6−528−A
C6−527−A
C6−526−A
C6−525−A
C6−524−A
C6−523−A
C6−522−A
C6−521−A
C6−520−A
C6−519−A
C6−518−A
C6−517−A
C6−516−A
C6−515−A
C6−514−A
C6−513−A
C6−512−A
C6−511−A
IDs
0
20
40
60
Mbps
Population
MNT_I
MNT_M

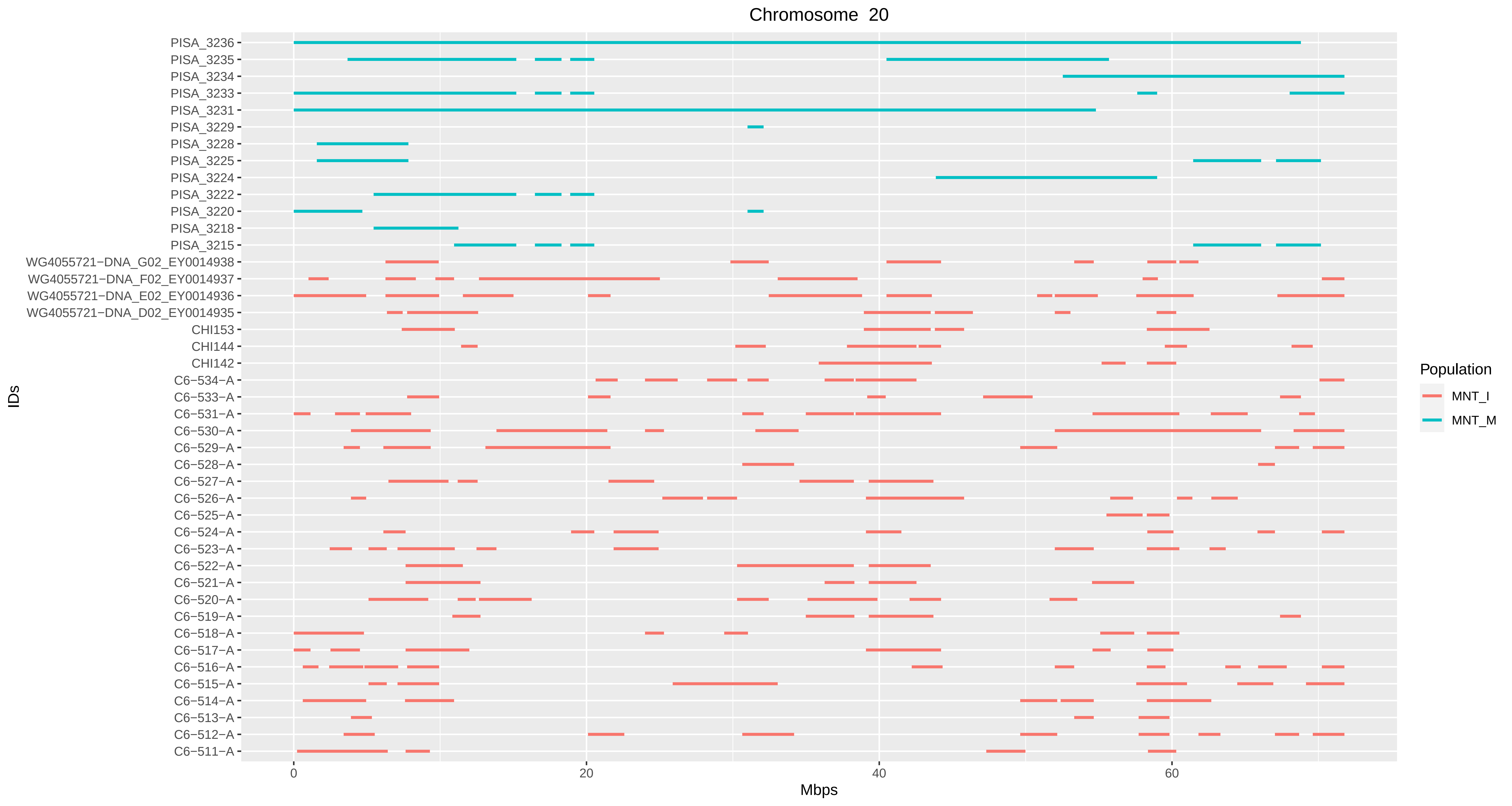

Chromosome 20
PISA_3236
PISA_3235
PISA_3234
PISA_3233
PISA_3231
PISA_3229
PISA_3228
PISA_3225
PISA_3224
PISA_3222
PISA_3220
PISA_3218
PISA_3215
WG4055721−DNA_G02_EY0014938
WG4055721−DNA_F02_EY0014937
WG4055721−DNA_E02_EY0014936
WG4055721−DNA_D02_EY0014935
CHI153
CHI144
CHI142
C6−534−A
C6−533−A
C6−531−A
C6−530−A
C6−529−A
C6−528−A
C6−527−A
C6−526−A
C6−525−A
C6−524−A
C6−523−A
C6−522−A
C6−521−A
C6−520−A
C6−519−A
C6−518−A
C6−517−A
C6−516−A
C6−515−A
C6−514−A
C6−513−A
C6−512−A
C6−511−A
IDs
0
20
40
60
Mbps
Population
MNT_I
MNT_M

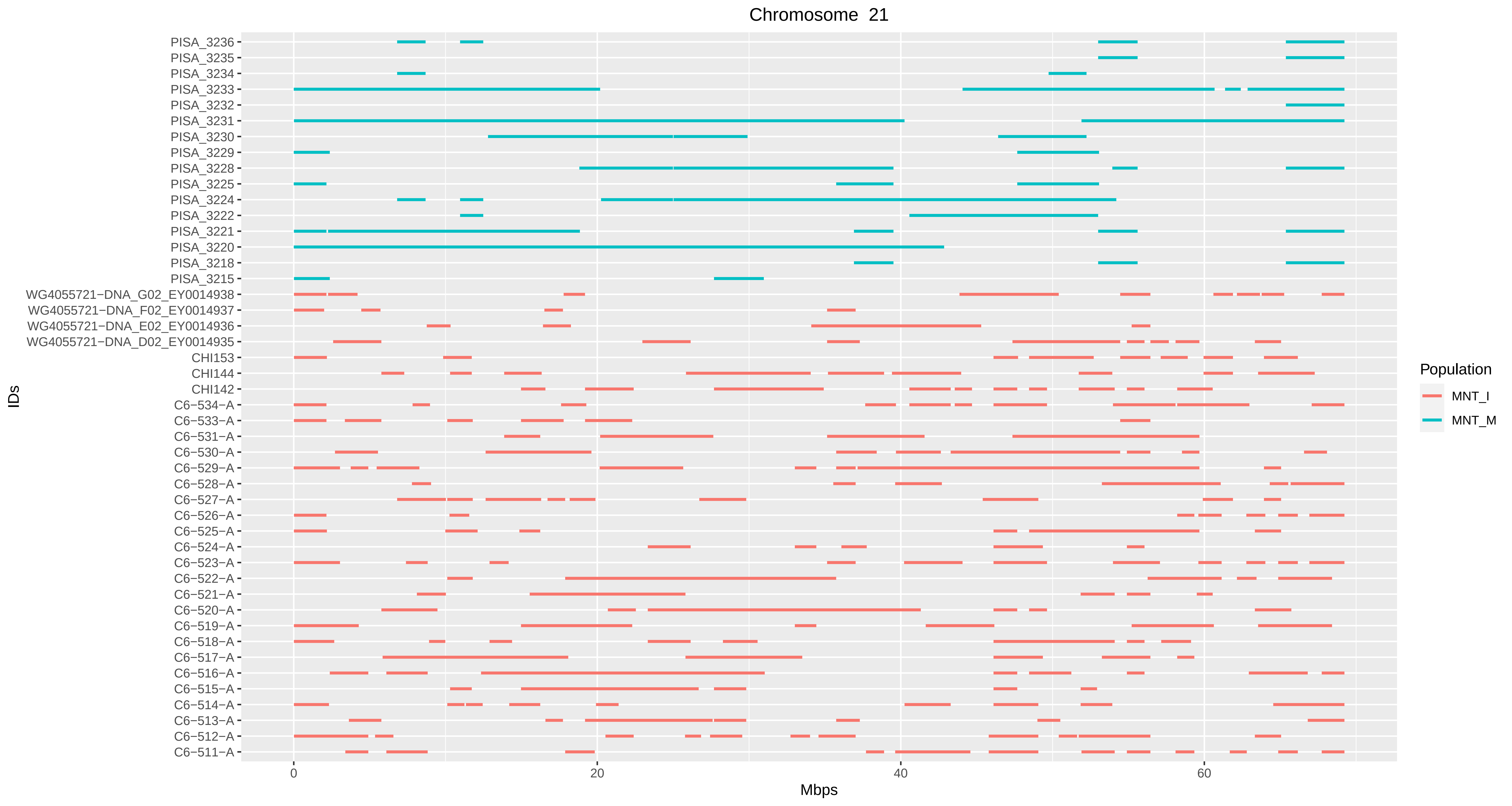

Chromosome 21
PISA_3236
PISA_3235
PISA_3234
PISA_3233
PISA_3232
PISA_3231
PISA_3230
PISA_3229
PISA_3228
PISA_3225
PISA_3224
PISA_3222
PISA_3221
PISA_3220
PISA_3218
PISA_3215
WG4055721−DNA_G02_EY0014938
WG4055721−DNA_F02_EY0014937
WG4055721−DNA_E02_EY0014936
WG4055721−DNA_D02_EY0014935
CHI153
CHI144
CHI142
C6−534−A
C6−533−A
C6−531−A
C6−530−A
C6−529−A
C6−528−A
C6−527−A
C6−526−A
C6−525−A
C6−524−A
C6−523−A
C6−522−A
C6−521−A
C6−520−A
C6−519−A
C6−518−A
C6−517−A
C6−516−A
C6−515−A
C6−514−A
C6−513−A
C6−512−A
C6−511−A
IDs
0
20
40
60
Mbps
Population
MNT_I
MNT_M

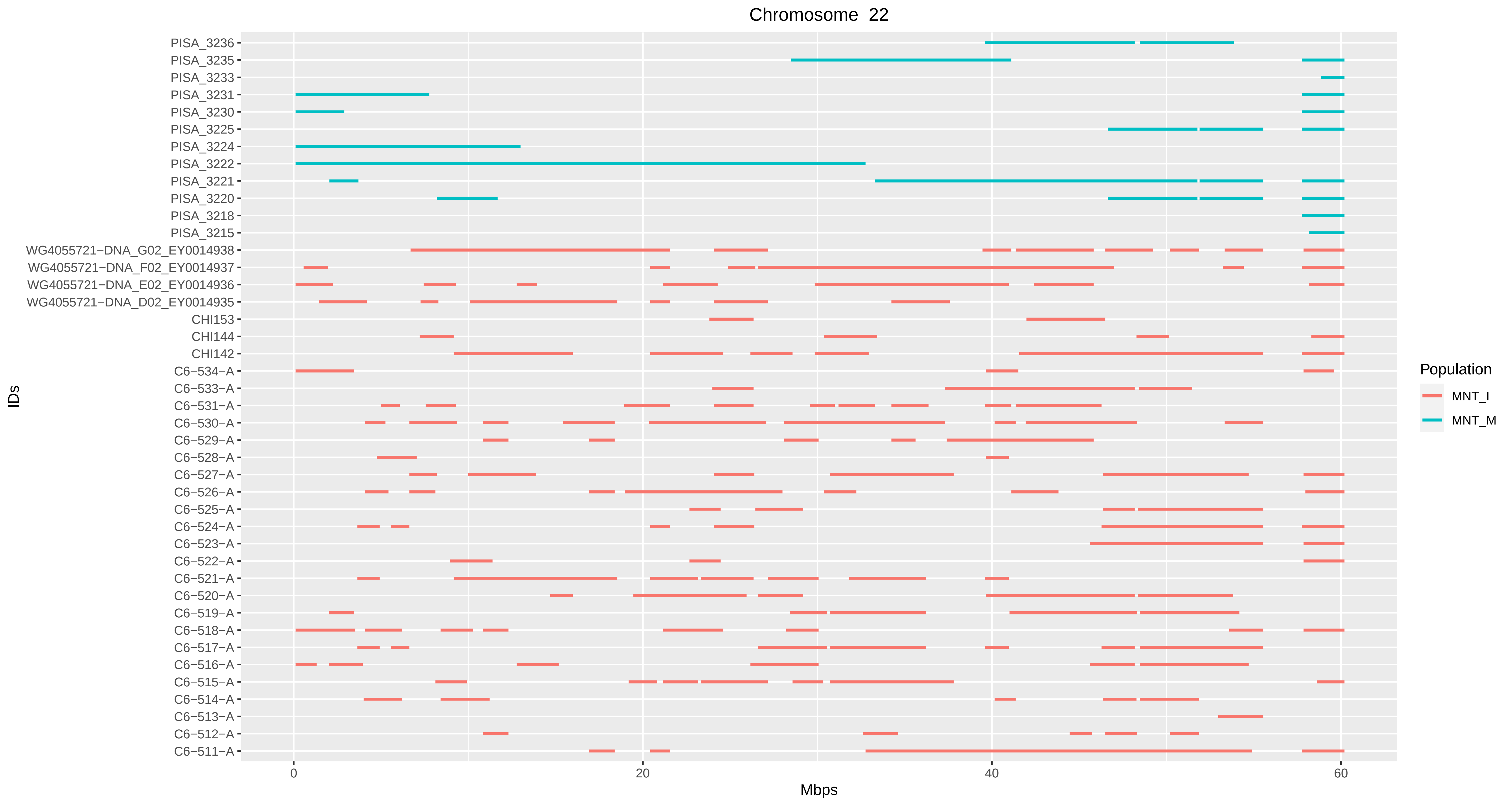
Chromosome 22
PISA_3236
PISA_3235
PISA_3233
PISA_3231
PISA_3230
PISA_3225
PISA_3224
PISA_3222
PISA_3221
PISA_3220
PISA_3218
PISA_3215
WG4055721−DNA_G02_EY0014938
WG4055721−DNA_F02_EY0014937
WG4055721−DNA_E02_EY0014936
WG4055721−DNA_D02_EY0014935
CHI153
CHI144
CHI142
C6−534−A
C6−533−A
C6−531−A
C6−530−A
C6−529−A
C6−528−A
C6−527−A
C6−526−A
C6−525−A
C6−524−A
C6−523−A
C6−522−A
C6−521−A
C6−520−A
C6−519−A
C6−518−A
C6−517−A
C6−516−A
C6−515−A
C6−514−A
C6−513−A
C6−512−A
C6−511−A
IDs
0
20
40
60
Mbps
Population
MNT_I
MNT_M

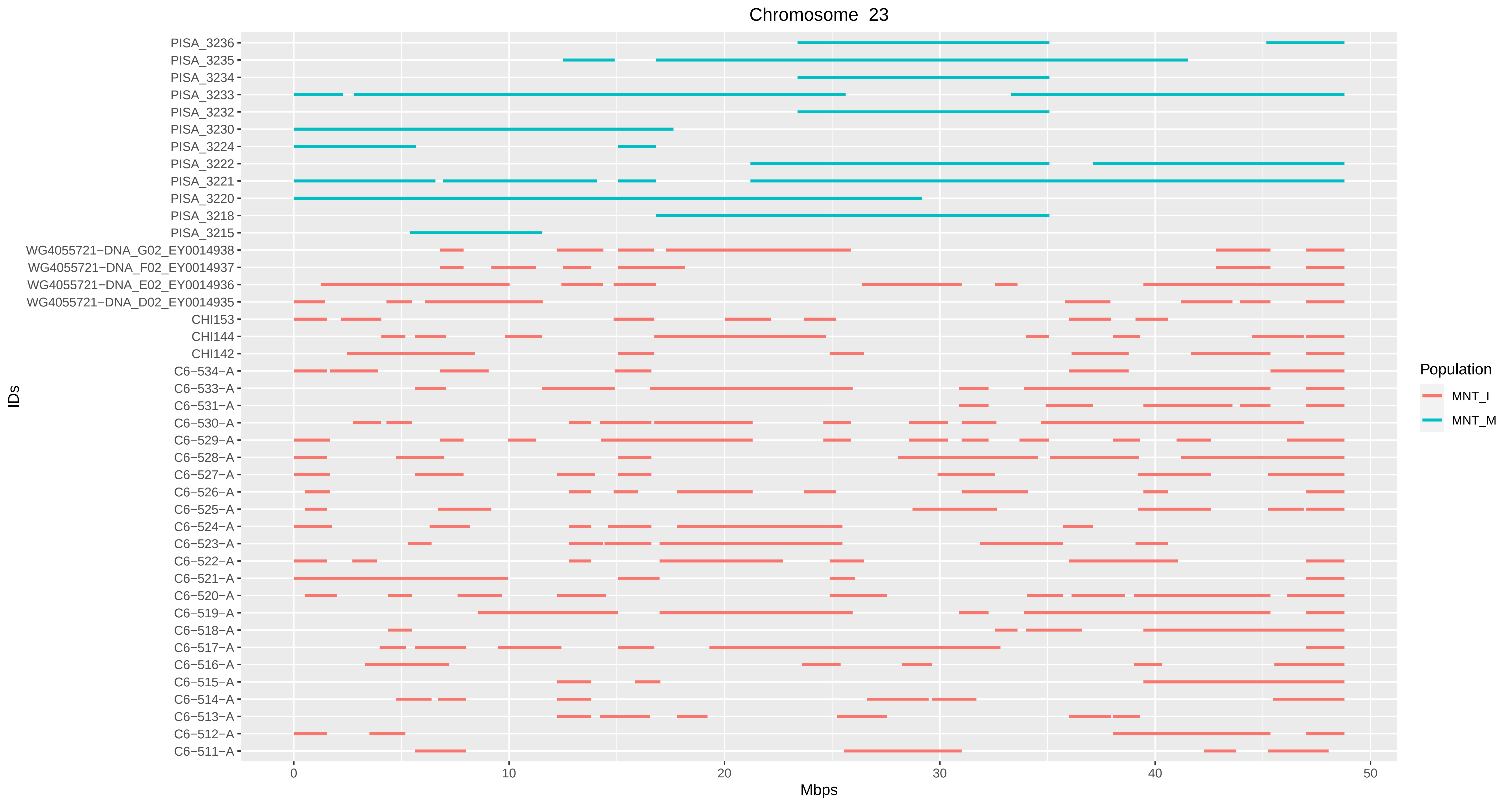

Chromosome 23
PISA_3236
PISA_3235
PISA_3234
PISA_3233
PISA_3232
PISA_3230
PISA_3224
PISA_3222
PISA_3221
PISA_3220
PISA_3218
PISA_3215
WG4055721−DNA_G02_EY0014938
WG4055721−DNA_F02_EY0014937
WG4055721−DNA_E02_EY0014936
WG4055721−DNA_D02_EY0014935
CHI153
CHI144
CHI142
C6−534−A
C6−533−A
C6−531−A
C6−530−A
C6−529−A
C6−528−A
C6−527−A
C6−526−A
C6−525−A
C6−524−A
C6−523−A
C6−522−A
C6−521−A
C6−520−A
C6−519−A
C6−518−A
C6−517−A
C6−516−A
C6−515−A
C6−514−A
C6−513−A
C6−512−A
C6−511−A
IDs
0
10
20
30
40
50
Mbps
Population
MNT_I
MNT_M

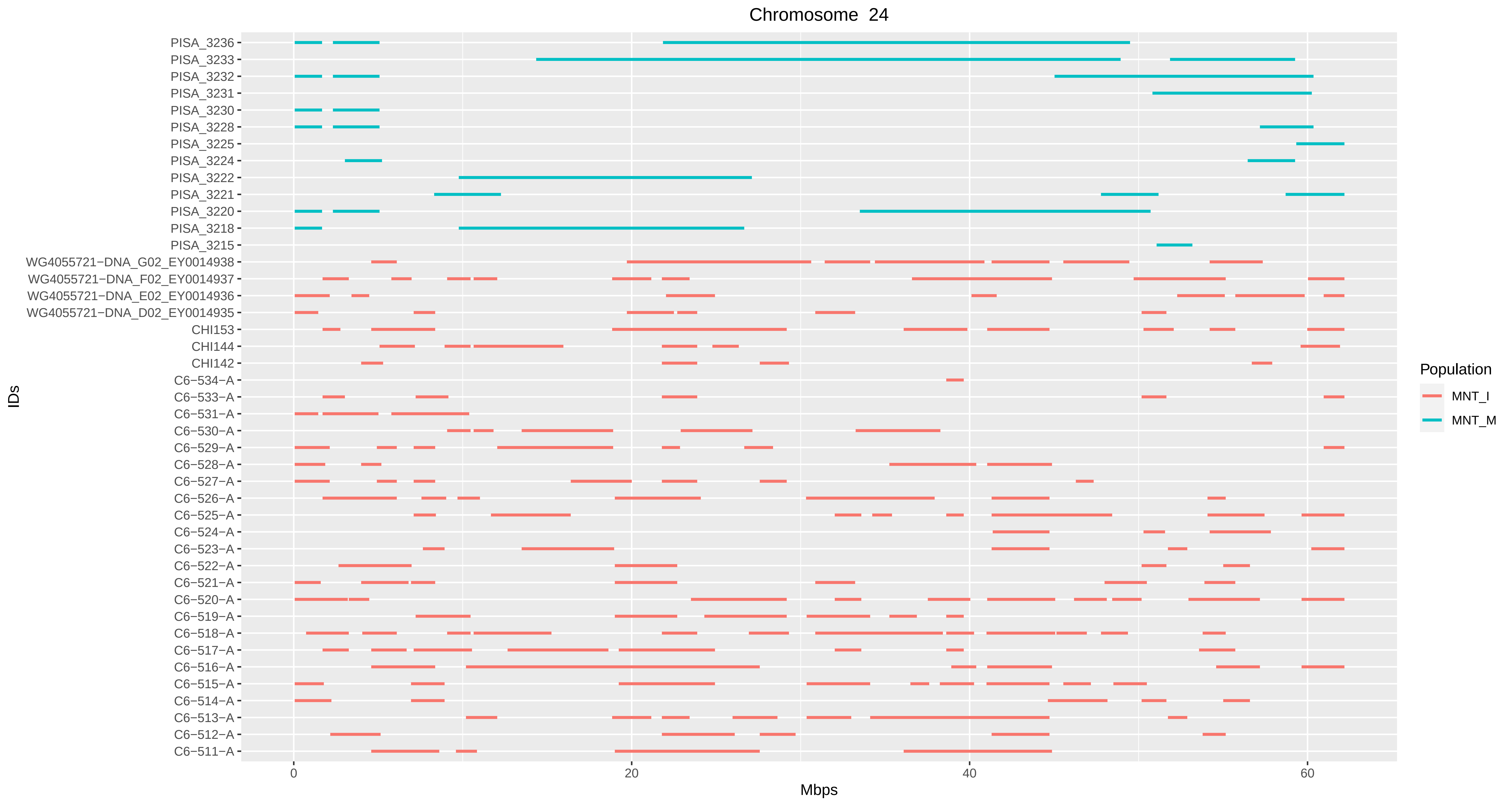

Chromosome 24
PISA_3236
PISA_3233
PISA_3232
PISA_3231
PISA_3230
PISA_3228
PISA_3225
PISA_3224
PISA_3222
PISA_3221
PISA_3220
PISA_3218
PISA_3215
WG4055721−DNA_G02_EY0014938
WG4055721−DNA_F02_EY0014937
WG4055721−DNA_E02_EY0014936
WG4055721−DNA_D02_EY0014935
CHI153
CHI144
CHI142
C6−534−A
C6−533−A
C6−531−A
C6−530−A
C6−529−A
C6−528−A
C6−527−A
C6−526−A
C6−525−A
C6−524−A
C6−523−A
C6−522−A
C6−521−A
C6−520−A
C6−519−A
C6−518−A
C6−517−A
C6−516−A
C6−515−A
C6−514−A
C6−513−A
C6−512−A
C6−511−A
IDs
0
20
40
60
Mbps
Population
MNT_I
MNT_M

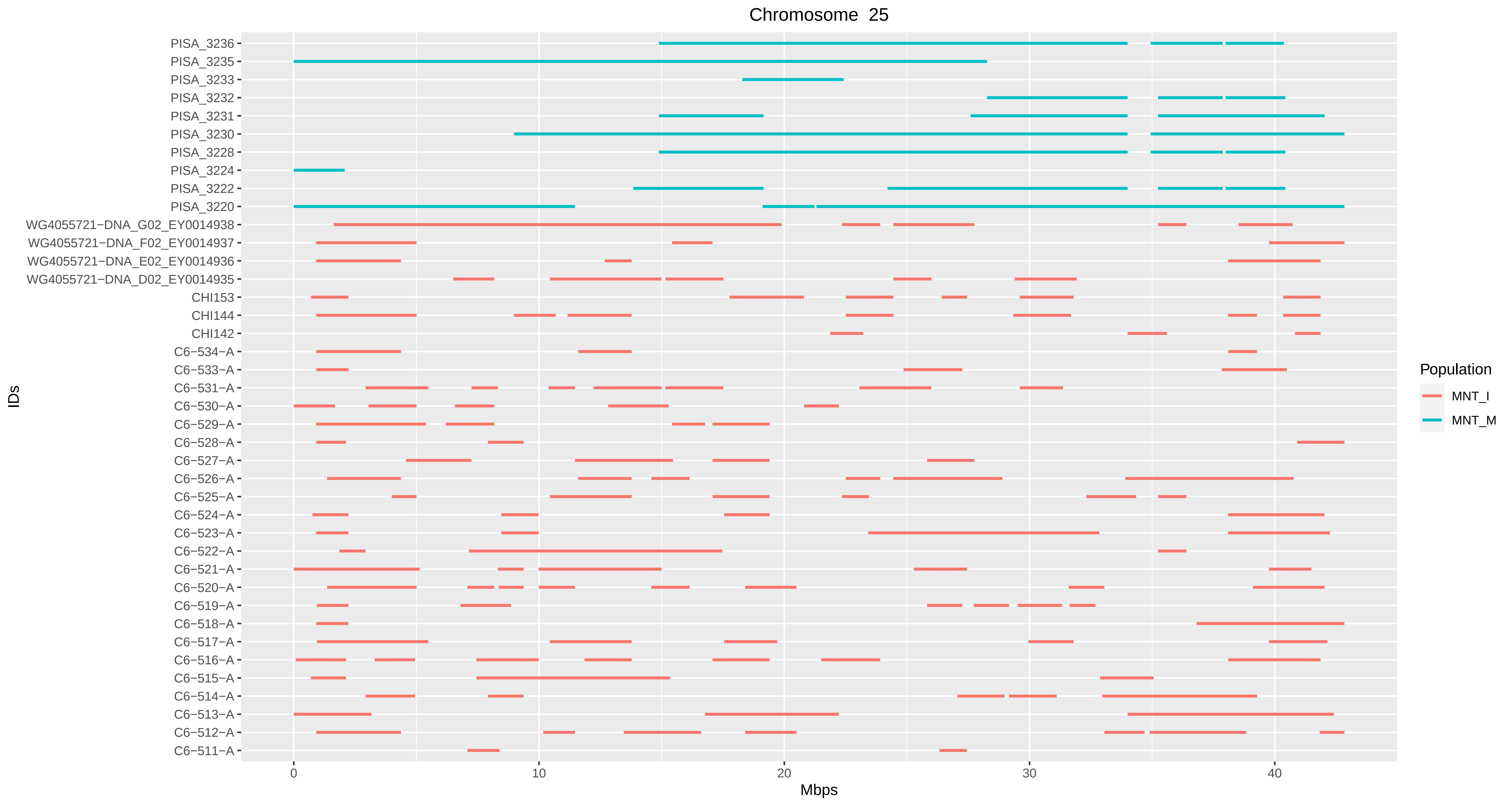

Chromosome 25
PISA_3236
PISA_3235
PISA_3233
PISA_3232
PISA_3231
PISA_3230
PISA_3228
PISA_3224
PISA_3222
PISA_3220
WG4055721−DNA_G02_EY0014938
WG4055721−DNA_F02_EY0014937
WG4055721−DNA_E02_EY0014936
WG4055721−DNA_D02_EY0014935
CHI153
CHI144
CHI142
C6−534−A
C6−533−A
C6−531−A
C6−530−A
C6−529−A
C6−528−A
C6−527−A
C6−526−A
C6−525−A
C6−524−A
C6−523−A
C6−522−A
C6−521−A
C6−520−A
C6−519−A
C6−518−A
C6−517−A
C6−516−A
C6−515−A
C6−514−A
C6−513−A
C6−512−A
C6−511−A
IDs
0
10
20
30
40
Mbps
Population
MNT_I
MNT_M

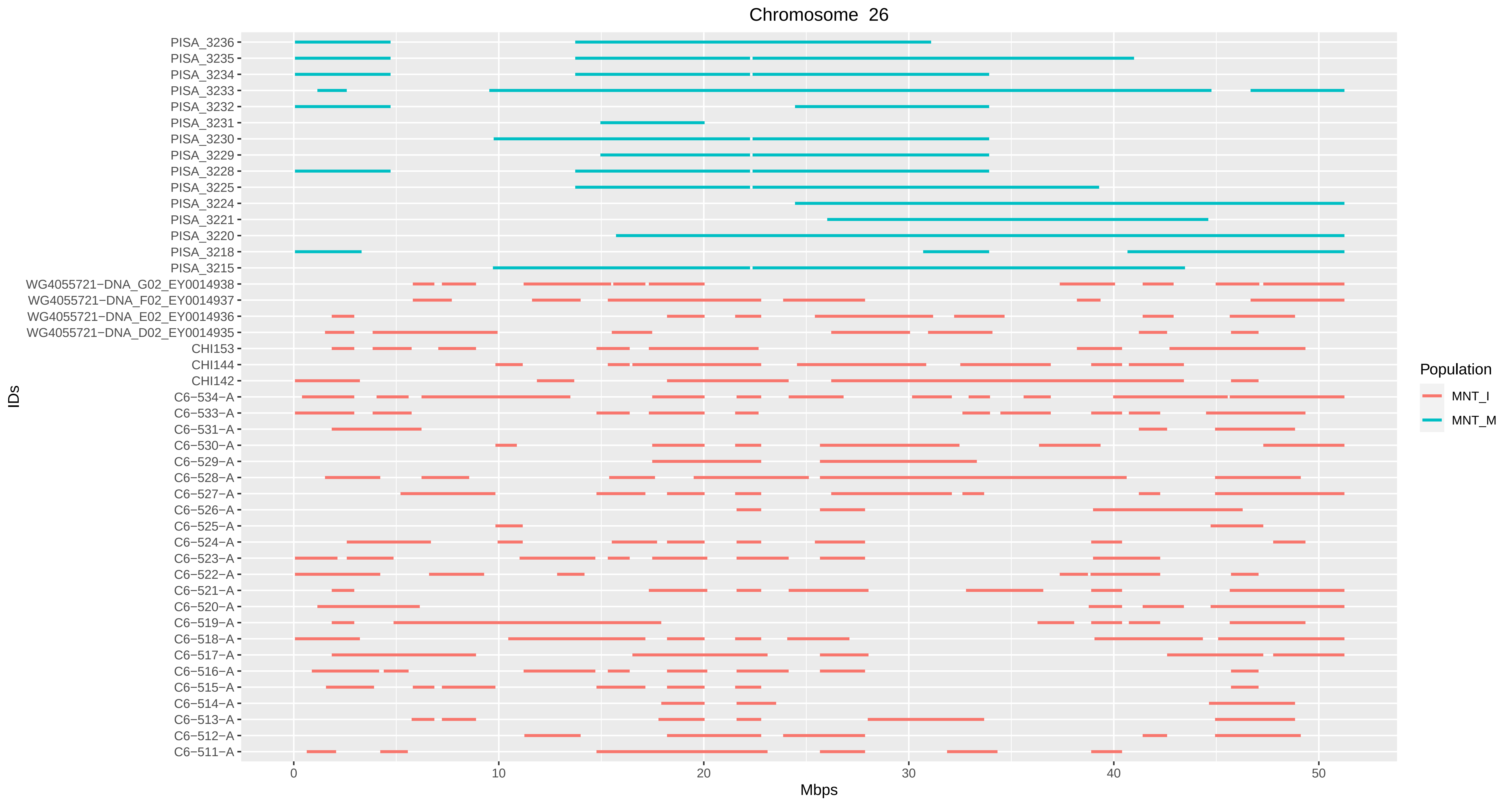

Chromosome 26
PISA_3236
PISA_3235
PISA_3234
PISA_3233
PISA_3232
PISA_3231
PISA_3230
PISA_3229
PISA_3228
PISA_3225
PISA_3224
PISA_3221
PISA_3220
PISA_3218
PISA_3215
WG4055721−DNA_G02_EY0014938
WG4055721−DNA_F02_EY0014937
WG4055721−DNA_E02_EY0014936
WG4055721−DNA_D02_EY0014935
CHI153
CHI144
CHI142
C6−534−A
C6−533−A
C6−531−A
C6−530−A
C6−529−A
C6−528−A
C6−527−A
C6−526−A
C6−525−A
C6−524−A
C6−523−A
C6−522−A
C6−521−A
C6−520−A
C6−519−A
C6−518−A
C6−517−A
C6−516−A
C6−515−A
C6−514−A
C6−513−A
C6−512−A
C6−511−A
IDs
0
10
20
30
40
50
Mbps
Population
MNT_I
MNT_M

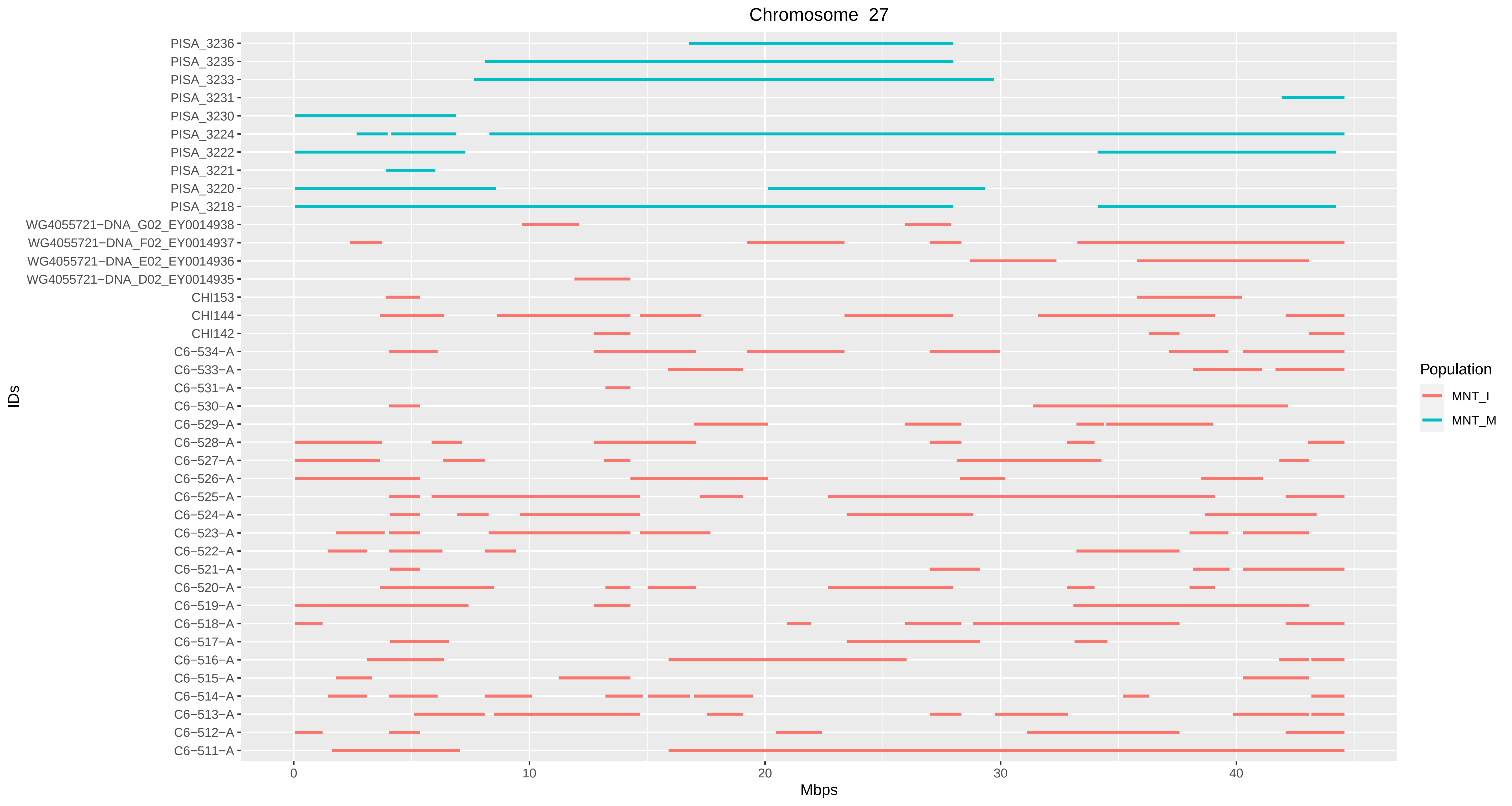
Chromosome 27
PISA_3236
PISA_3235
PISA_3233
PISA_3231
PISA_3230
PISA_3224
PISA_3222
PISA_3221
PISA_3220
PISA_3218
WG4055721−DNA_G02_EY0014938
WG4055721−DNA_F02_EY0014937
WG4055721−DNA_E02_EY0014936
WG4055721−DNA_D02_EY0014935
CHI153
CHI144
CHI142
C6−534−A
C6−533−A
C6−531−A
C6−530−A
C6−529−A
C6−528−A
C6−527−A
C6−526−A
C6−525−A
C6−524−A
C6−523−A
C6−522−A
C6−521−A
C6−520−A
C6−519−A
C6−518−A
C6−517−A
C6−516−A
C6−515−A
C6−514−A
C6−513−A
C6−512−A
C6−511−A
IDs
0
10
20
30
40
Mbps
Population
MNT_I
MNT_M

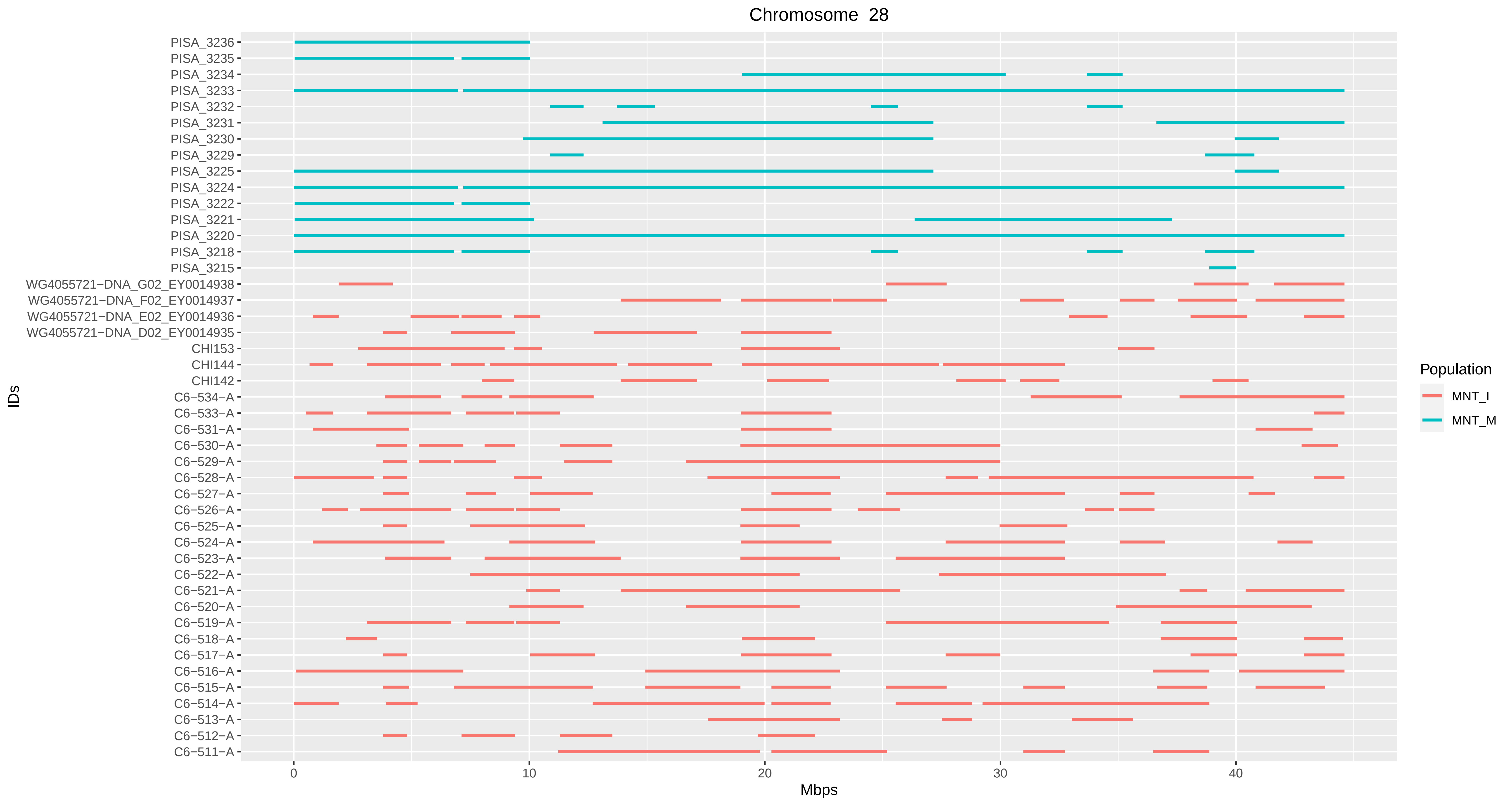

Chromosome 28
PISA_3236
PISA_3235
PISA_3234
PISA_3233
PISA_3232
PISA_3231
PISA_3230
PISA_3229
PISA_3225
PISA_3224
PISA_3222
PISA_3221
PISA_3220
PISA_3218
PISA_3215
WG4055721−DNA_G02_EY0014938
WG4055721−DNA_F02_EY0014937
WG4055721−DNA_E02_EY0014936
WG4055721−DNA_D02_EY0014935
CHI153
CHI144
CHI142
C6−534−A
C6−533−A
C6−531−A
C6−530−A
C6−529−A
C6−528−A
C6−527−A
C6−526−A
C6−525−A
C6−524−A
C6−523−A
C6−522−A
C6−521−A
C6−520−A
C6−519−A
C6−518−A
C6−517−A
C6−516−A
C6−515−A
C6−514−A
C6−513−A
C6−512−A
C6−511−A
IDs
0
10
20
30
40
Mbps
Population
MNT_I
MNT_M

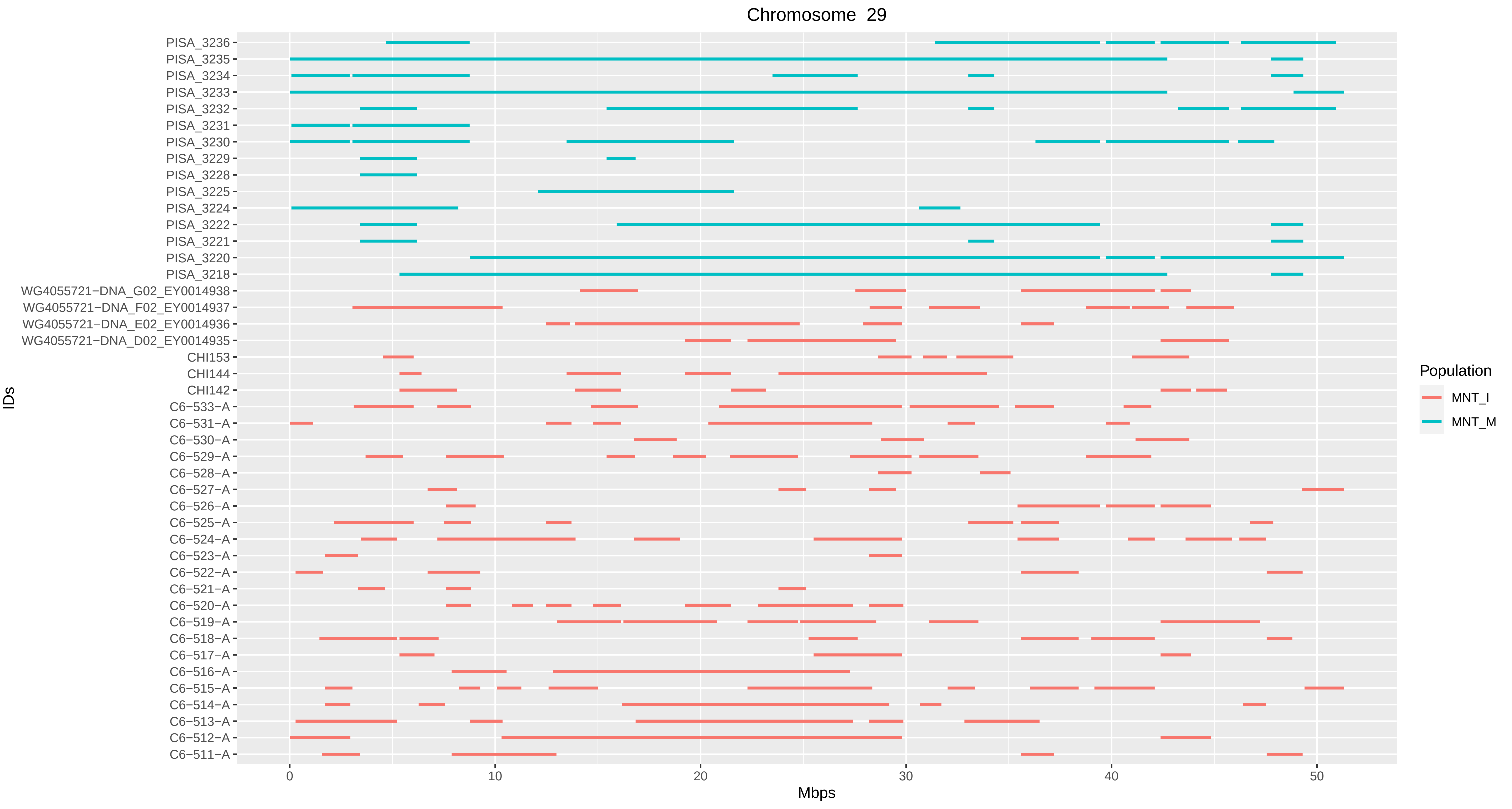
Chromosome 29
PISA_3236
PISA_3235
PISA_3234
PISA_3233
PISA_3232
PISA_3231
PISA_3230
PISA_3229
PISA_3228
PISA_3225
PISA_3224
PISA_3222
PISA_3221
PISA_3220
PISA_3218
WG4055721−DNA_G02_EY0014938
WG4055721−DNA_F02_EY0014937
WG4055721−DNA_E02_EY0014936
WG4055721−DNA_D02_EY0014935
CHI153
CHI144
CHI142
C6−533−A
C6−531−A
C6−530−A
C6−529−A
C6−528−A
C6−527−A
C6−526−A
C6−525−A
C6−524−A
C6−523−A
C6−522−A
C6−521−A
C6−520−A
C6−519−A
C6−518−A
C6−517−A
C6−516−A
C6−515−A
C6−514−A
C6−513−A
C6−512−A
C6−511−A
IDs
0
10
20
30
40
50
Mbps
Population
MNT_I
MNT_M
