# Supplementary figures and images for: The SNP-Based Profiling of Montecristo Feral Goat Populations Reveals a History of Isolation, Bottlenecks, and the Effects of Management
Source: Genes (Basel). 2022 Jan 24;13(2):213. doi: 10.3390/genes13020213 (PMC8872249; doi:10.3390/genes13020213)

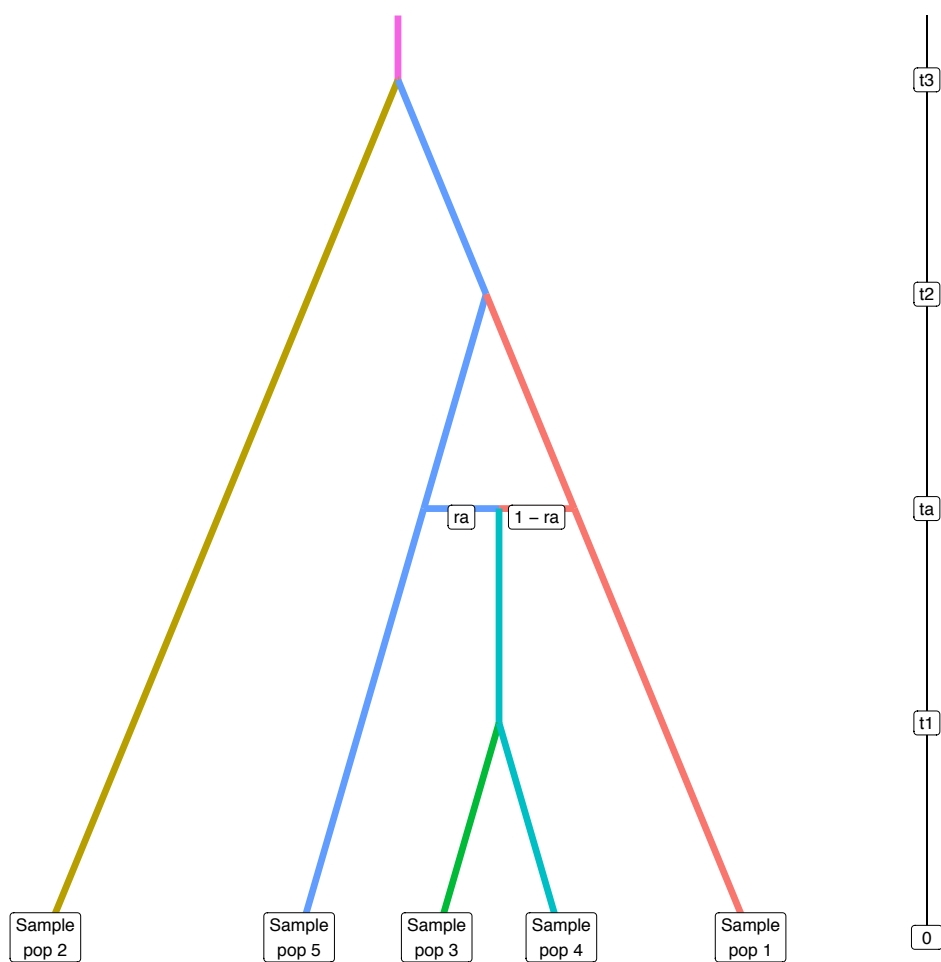

(a)

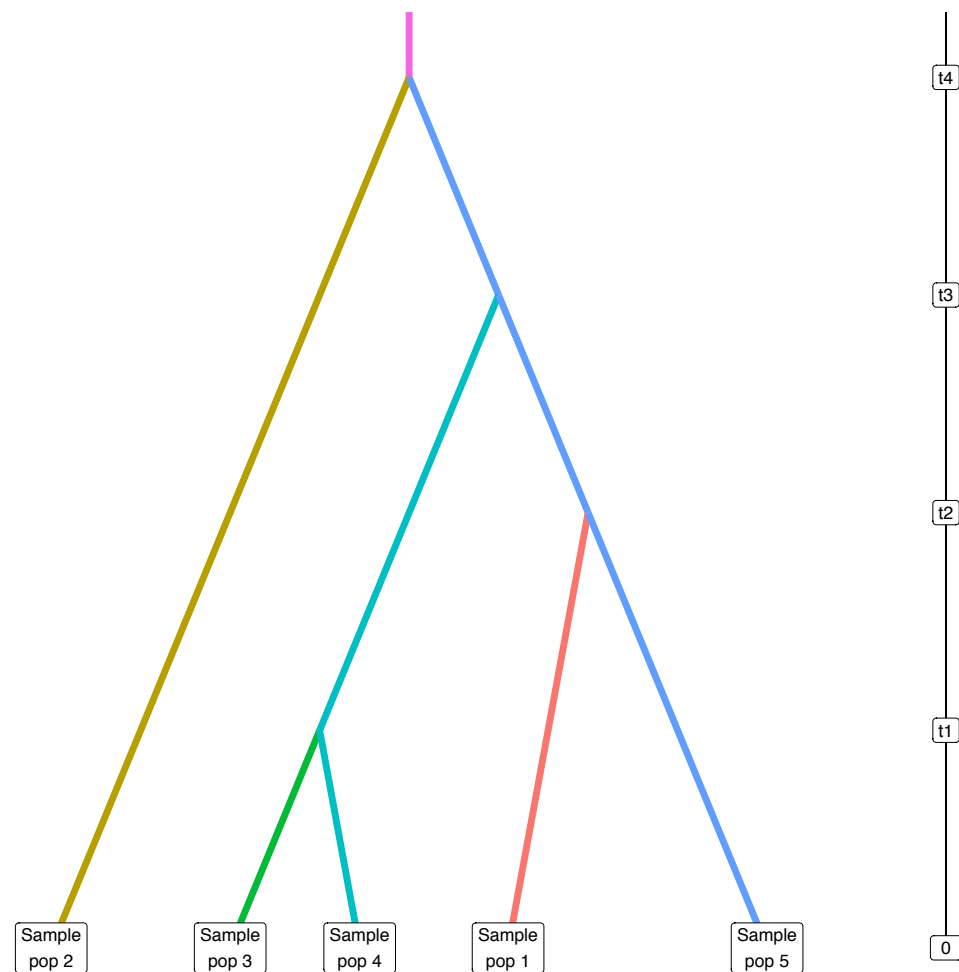

(b)

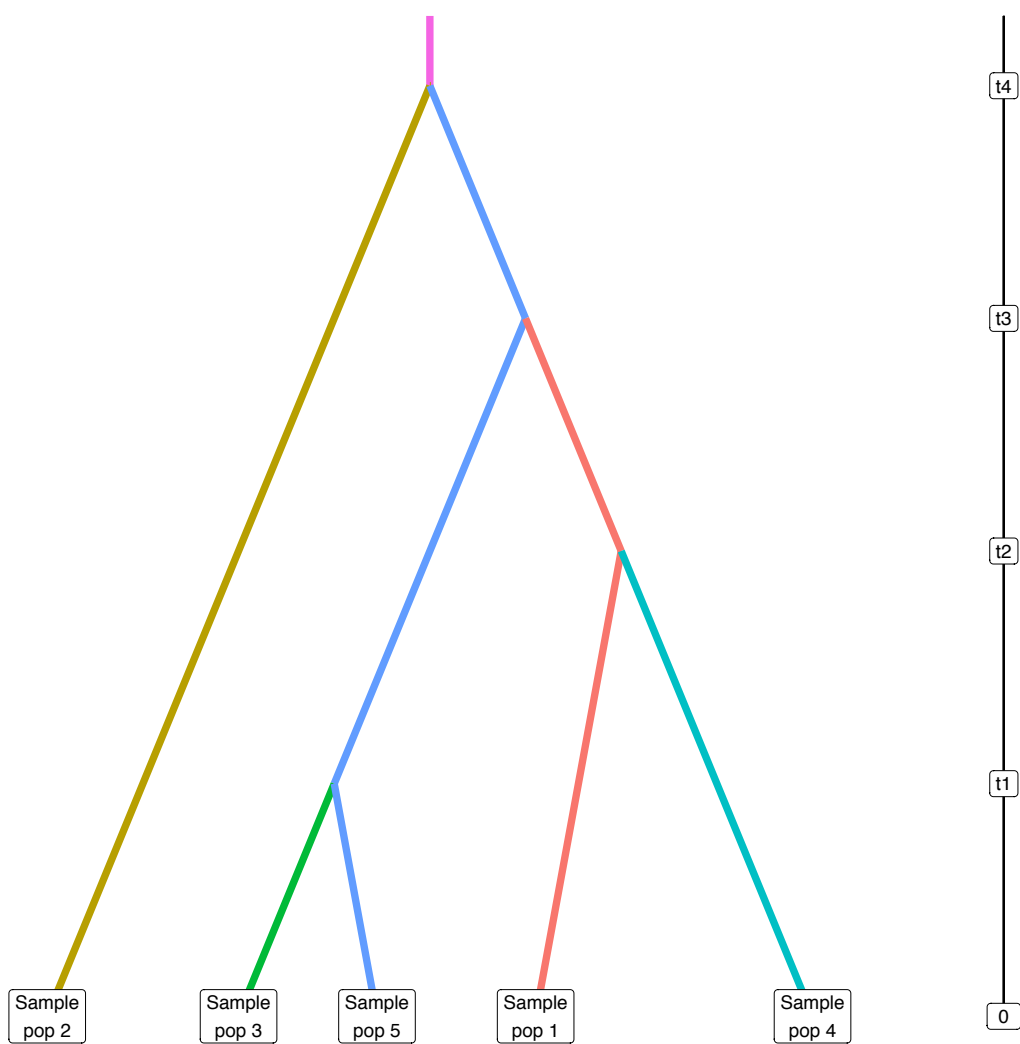

(c)

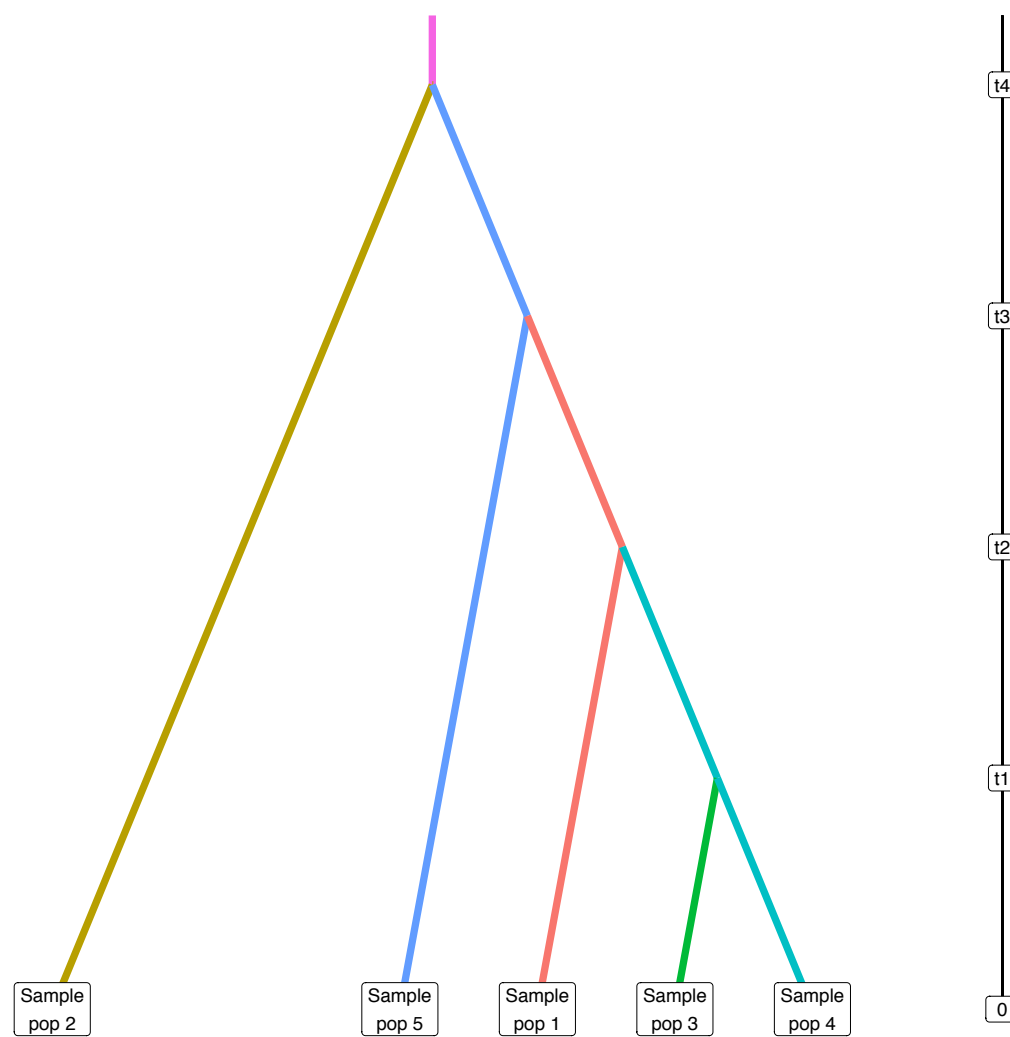

(d)

Supplement: Supplementary file 1 [file genes-13-00213-s001.zip › SFigure1.pdf]

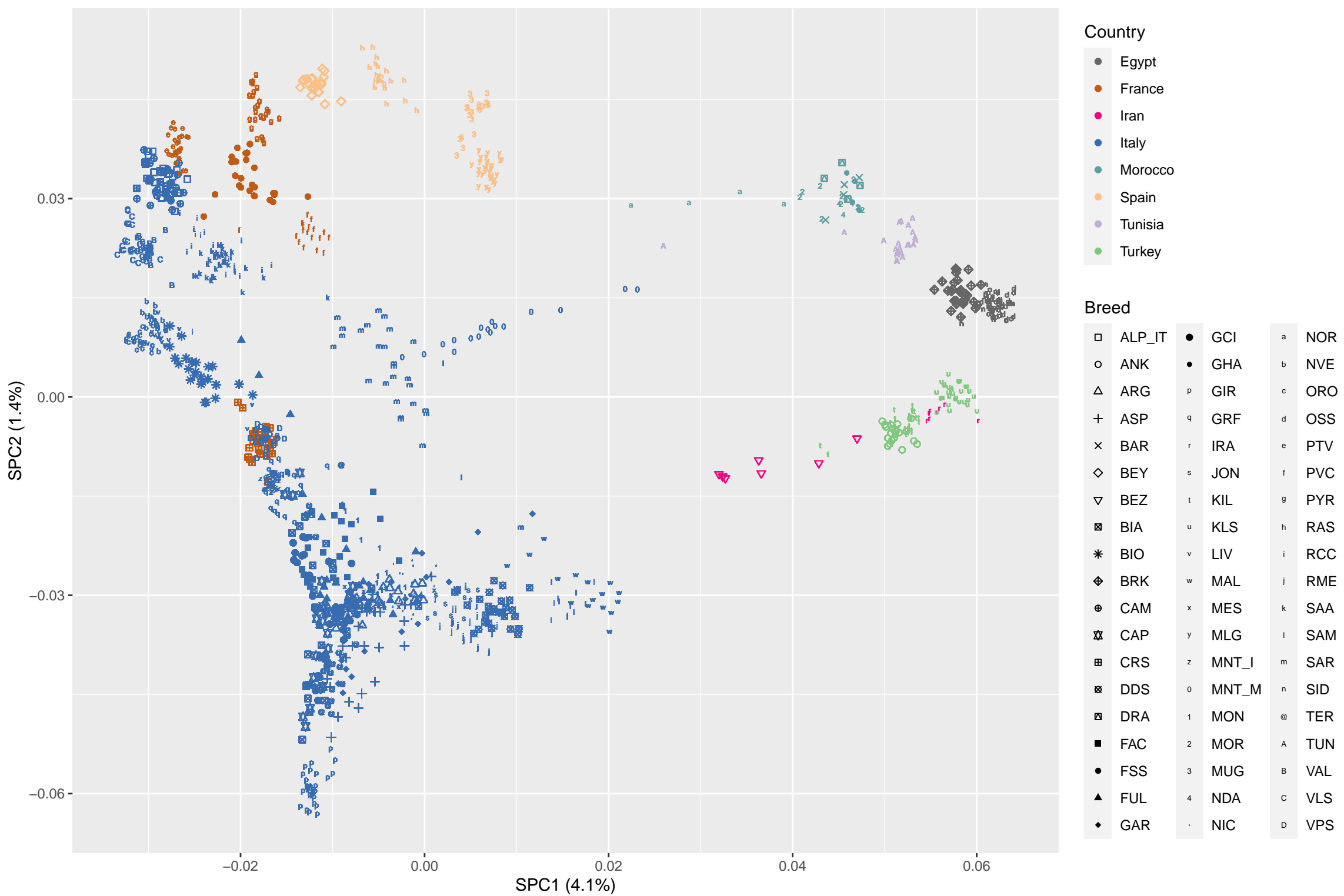

Supplement: Supplementary file 1 [file genes-13-00213-s001.zip › SFigure2.pdf]

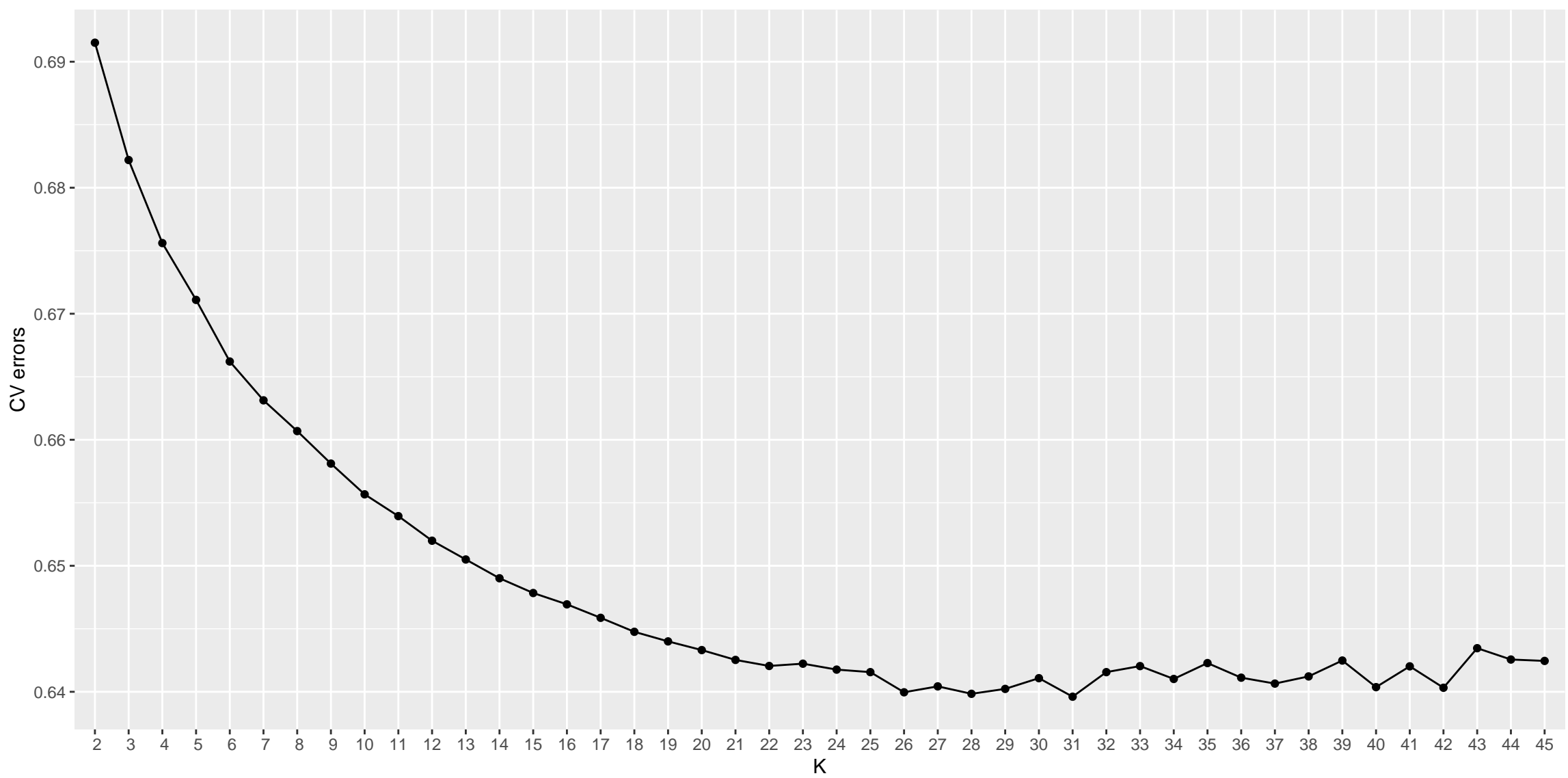

Supplement: Supplementary file 1 [file genes-13-00213-s001.zip › SFigure3.pdf]

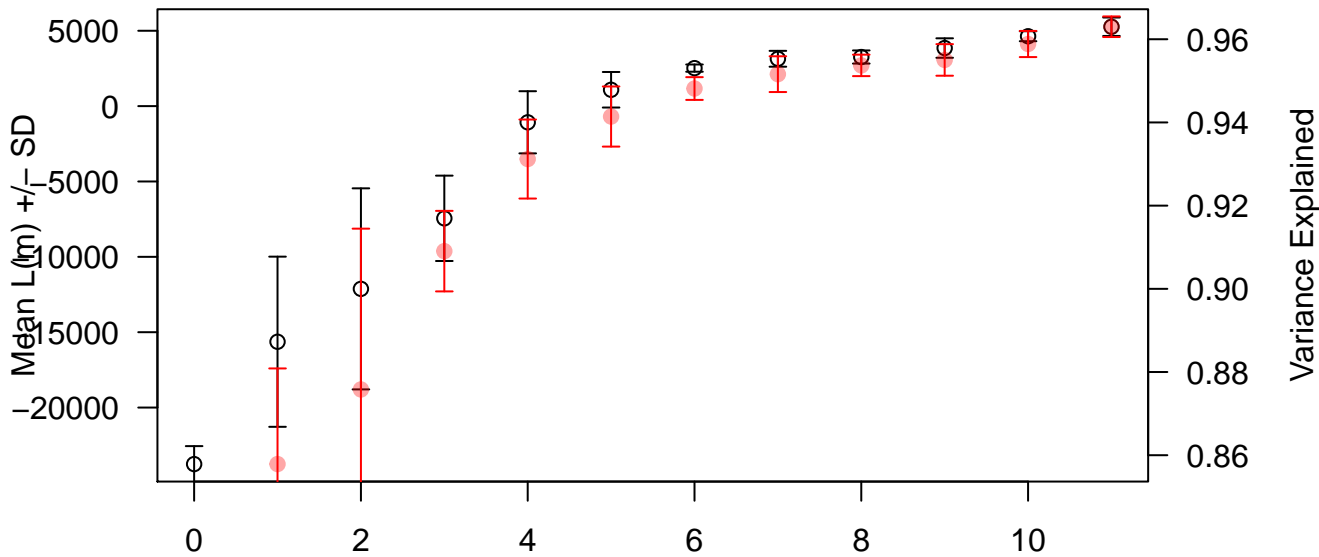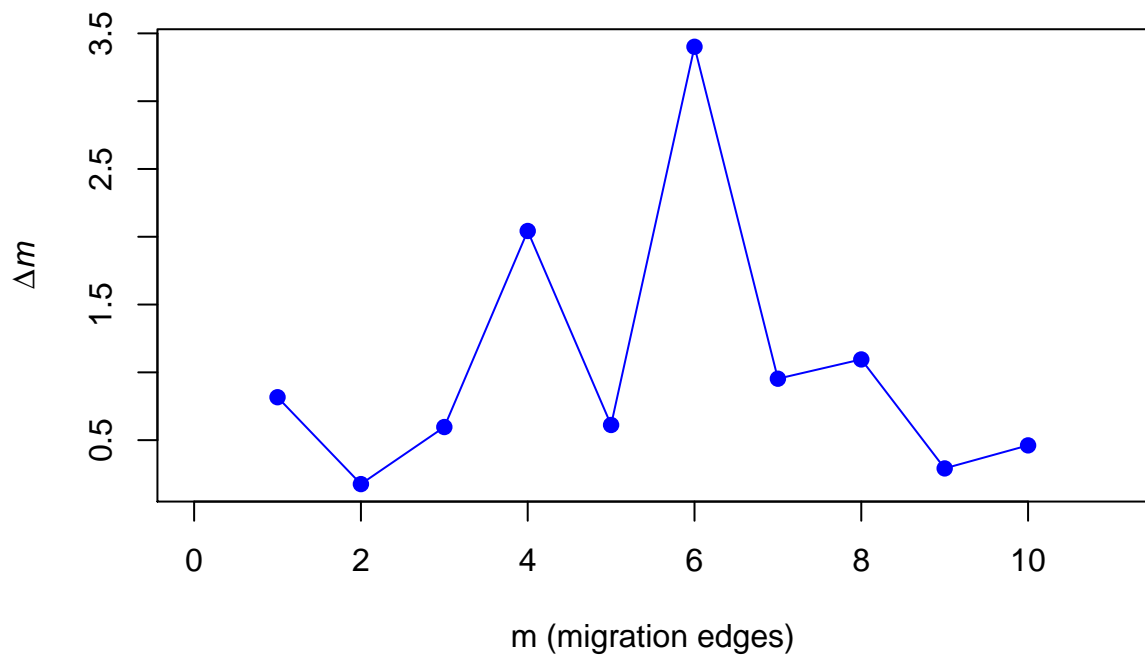

Supplement: Supplementary file 1 [file genes-13-00213-s001.zip › SFigure4.pdf]

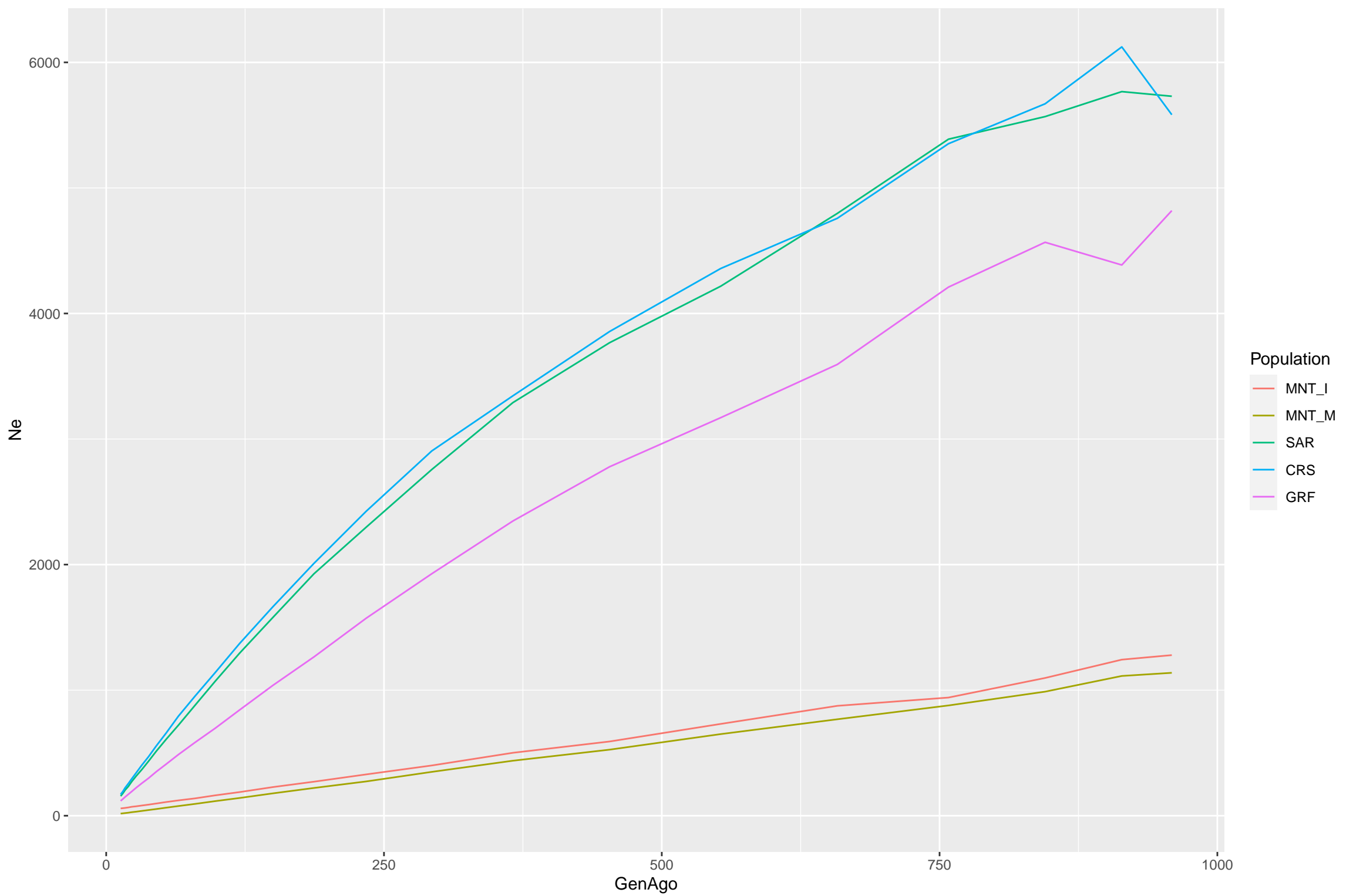

Supplement: Supplementary file 1 [file genes-13-00213-s001.zip › SFigure5.pdf]

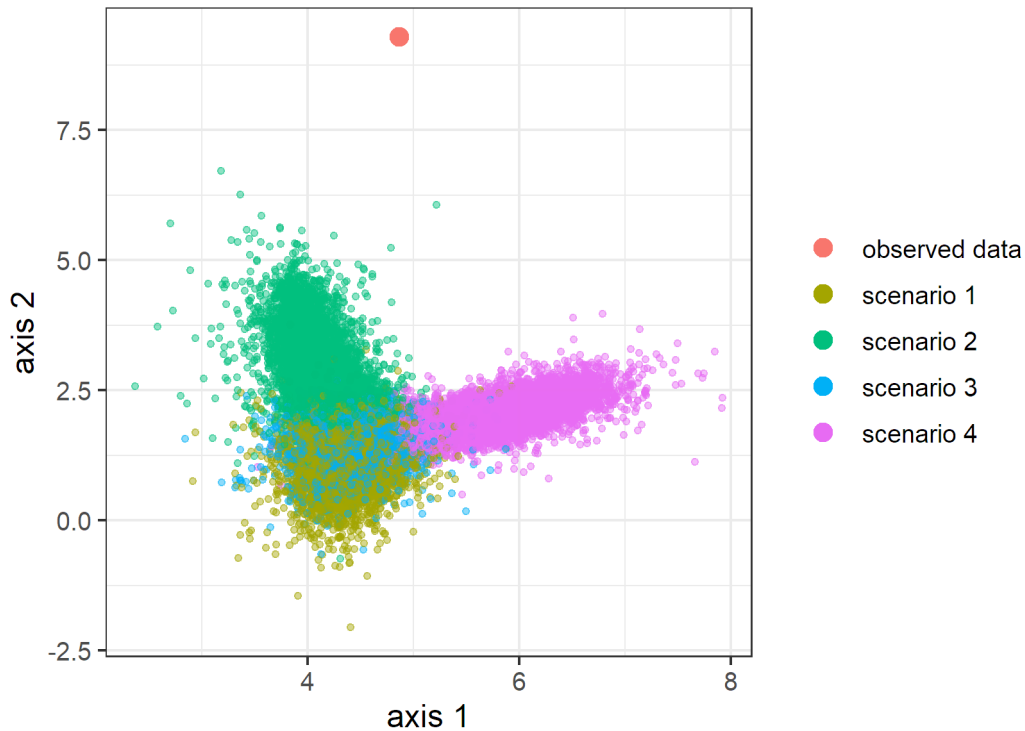

Supplement: Supplementary file 1 [file genes-13-00213-s001.zip › SFigure6.pdf]
